# Rb and FZR1/Cdh1 determine CDK4/6-cyclin D requirement in *C. elegans* and human cancer cells

**DOI:** 10.1038/ncomms6906

**Published:** 2015-01-06

**Authors:** Inge The, Suzan Ruijtenberg, Benjamin P. Bouchet, Alba Cristobal, Martine B. W. Prinsen, Tim van Mourik, John Koreth, Huihong Xu, Albert J. R. Heck, Anna Akhmanova, Edwin Cuppen, Mike Boxem, Javier Muñoz, Sander van den Heuvel

**Affiliations:** 1 Developmental Biology, Faculty of Sciences, Department of Biology, Utrecht University, Padualaan 8, 3584 CH Utrecht, The Netherlands; 2 Cell Biology, Faculty of Sciences, Department of Biology, Utrecht University, Padualaan 8, 3584 CH, Utrecht, The Netherlands; 3 Biomolecular Mass Spectrometry and Proteomics Group, Bijvoet Center for Biomolecular Research and Utrecht Institute for Pharmaceutical Sciences, Utrecht University, Padualaan 8, 3584 CH Utrecht, The Netherlands; 4 Hematologic Oncology, Dana-Farber Cancer Institute, 450 Brookline Avenue, Boston, Massachusetts 02215, USA; 5 Department of Pathology and Laboratory Medicine, Boston University School of Medicine and Boston Medical Center, 670 Albany Street, Boston, MA, USA; 6 Hubrecht Institute, Uppsalalaan 8, 3584 CT Utrecht, The Netherlands

## Abstract

Cyclin-dependent kinases 4 and 6 (CDK4/6) in complex with D-type cyclins promote cell cycle entry. Most human cancers contain overactive CDK4/6-cyclin D, and CDK4/6-specific inhibitors are promising anti-cancer therapeutics. Here, we investigate the critical functions of CDK4/6-cyclin D kinases, starting from an unbiased screen in the nematode *Caenorhabditis elegans.* We found that simultaneous mutation of *lin-35*, a retinoblastoma (Rb)-related gene, and *fzr-1*, an orthologue to the APC/C co-activator Cdh1, completely eliminates the essential requirement of CDK4/6-cyclin D (CDK-4/CYD-1) in *C. elegans*. CDK-4/CYD-1 phosphorylates specific residues in the LIN-35 Rb spacer domain and FZR-1 amino terminus, resembling inactivating phosphorylations of the human proteins. In human breast cancer cells, simultaneous knockdown of Rb and FZR1 synergistically bypasses cell division arrest induced by the CDK4/6-specific inhibitor PD-0332991. Our data identify FZR1 as a candidate CDK4/6-cyclin D substrate and point to an APC/C^FZR1^ activity as an important determinant in response to CDK4/6-inhibitors.

## Abstract (web summary)

In most human tumours, the cell cycle regulators Cdk4/6-cyclinD are overactive. Here the authors use *C. elegans* as a model system to identify downstream regulators that are critical in the response of tumour cells to Cdk4/6 inhibitors.

## Graphical abstract

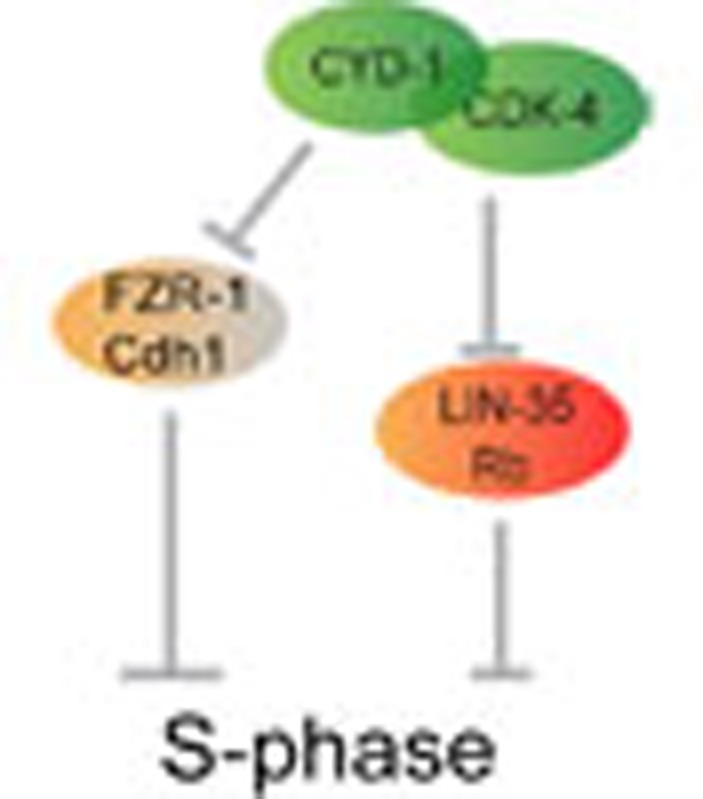

Cyclin-dependent kinases (CDKs) in association with cyclin subunits are the key regulators of cell division in eukaryotes^1^. Active CDK-cyclin complexes promote progression through the cell division cycle by phosphorylating critical substrates. The decision to initiate cell division occurs in the G1 phase and involves expression of D-type cyclins in response to mitogenic signalling^2^. These cyclins assemble with CDK4 or CDK6 (CDK4/6), to form kinase complexes that are best known for their phosphorylation of the retinoblastoma (Rb) tumour suppressor protein. Rb family proteins associate with E2F transcription factors and act as transcriptional repressors of cyclin E and other S phase genes. The initial phosphorylation of Rb by CDK4/6-cyclin D is thought to weaken its repressor function, while subsequent phosphorylation by CDK2-cyclin E completely inactivates Rb function and allows full commitment to S phase entry.

Strong support for the *in vivo* importance of this cell cycle control pathway has come from human cancer studies. Overexpression of CDK4/6 or D-type cyclins and inactivation of the CDK4/6 antagonist p16^INK4A^/CDKN2A or Rb tumour suppressor are common in human cancer^3^. These events are largely mutually exclusive, in support of p16^INK4A^, CDK4/6, D-type cyclins and Rb acting in a single regulatory pathway. At the same time, various studies have indicated that phosphorylation of Rb is not the only catalytic activity of CDK4/6-cyclin D kinases^4^,^5^. Additional substrates of cyclin D kinases have been described, the best characterized of which are the Rb-related proteins p107 and p130, and transcription factors SMAD3 and FOXM1 (refs 2, 4, 6). To what extent phosphorylation of these targets contributes to carcinogenesis is currently unknown.

Results from studies in mice have caused doubt on whether the functions of CDK4/6-cyclin D kinases are essential for proliferation. Knockout of a single D-type cyclin gene causes limited defects and mice that lack all three D-type cyclins still develop until mid-to-late gestation^7^. Similarly, CDK4/CDK6 double knockout mice complete organogenesis and extensive cell proliferation, with death due to anaemia occurring only in the late stages of embryogenesis^8^. In contrast to normal development, cancer formation in various mouse models depends strongly on CDK4/6-cyclin D kinase activity^9^,^10^,^11^,^12^. This difference in requirement appears to provide a window of opportunity for therapeutics that block cancer growth, while sparing normal cells. Small molecule inhibitors with high specificity for CDK4/6 have been identified, with PD-0332991 as the leading example^13^,^14^. PD-0332991 induces proliferation arrest in a substantial subset of human cancer cell lines and inhibits cancer formation in mouse models^10^,^11^,^13^,^15^. Based on these results and recent Phase II and Phase III clinical trials, CDK4/6 inhibitors currently receive much attention as promising anti-cancer therapeutics^16^,^17^,^18^. Although there are substantially increased progression-free survival rates of cancer patient populations in several studies, biomarkers that predict a positive response to CDK4/6 inhibitor treatment are currently not known. It will be of great clinical importance to reveal which cancer genotypes correspond to cell cycle arrest, or even senescence and apoptosis, in response to inhibitor treatment, and which bypass routes may be used by cancer cells to acquire resistance to CDK4/6-specific inhibitors.

In this study, we examine the critical functions of the CDK4/6 cyclin D kinase, making use of the evolutionary conserved regulation of cell cycle entry in metazoans. Our observations in the nematode *C. elegans* support that Rb-mediated transcriptional repression and APC^FZR1^-mediated protein degradation act in parallel to inhibit G1/S progression, and that phosphorylation by the CDK-4/CYD-1 cyclin D kinase counteracts these inhibitory functions. Importantly, we also observed synergy between Rb and FZR1 knockdown in bypassing the proliferation arrest induced by treatment of human breast cancer cells with the CDK4/6 inhibitor PD-0332991. Our results indicate that the level of APC/C^FZR1^ activity is an important contributing factor in response of cancer cells to CDK4/6 inhibitor treatment.

## Results

### *C. elegans* CDK-4/CYD-1 has multiple critical substrates

**Figure 1:**
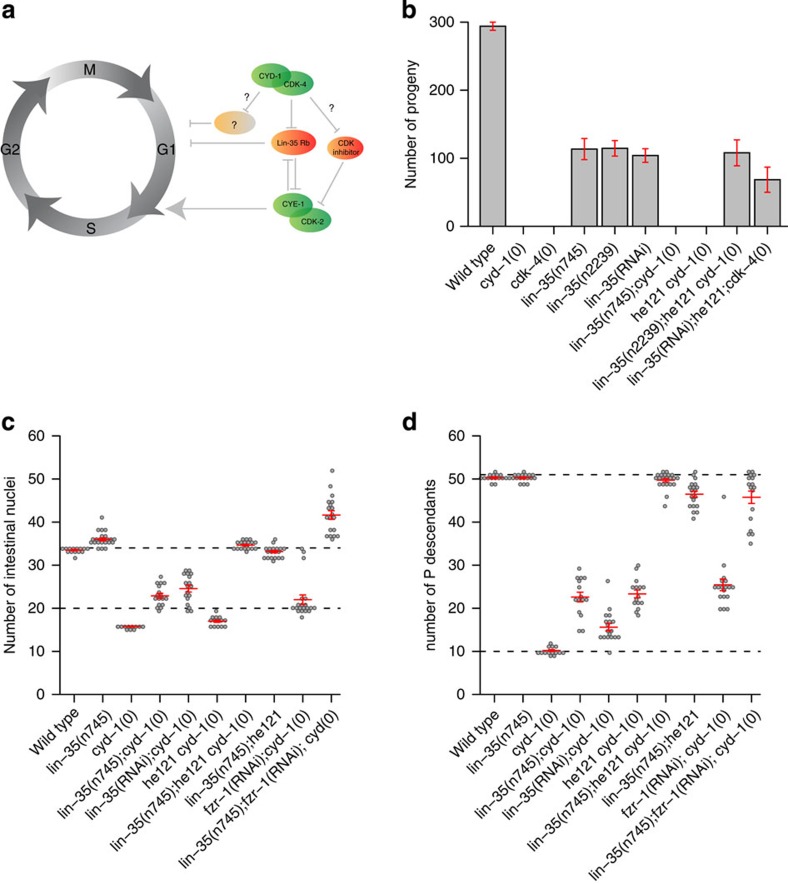
Simultaneous *lin-35* Rb and *fzr-1* loss of function eliminates *cyd-1* and *cdk-4* requirement. (**a**) Model for cell cycle entry in *C. elegans* illustrating more than one critical function of CYD-1/CDK-4. (**b**) Quantification of total progeny numbers (bars) for animals of the indicated genotypes. (**c**) Quantification of the number of intestinal nuclei for the indicated genotypes. (**d**) Quantification of the number of descendants of the *P*_2_–*P*_10_ ventral cord precursor cells for the indicated genotypes. The candidate-null alleles used are *cyd-1(he112)*, *cdk-4(gv3)* and the *lin-35* alleles *n745* and *n2239.* Total broods were counted of at least two animals of each genotype and total cell numbers in the indicated tissues of at least ten animals of each genotype. Graphs show mean±s.e.m., each dot (**c**,**d**) represents a single animal.

We followed a genetic approach to reveal critical functions of CDK4/6 kinases. Cell cycle entry in *C. elegans* involves a CDK4/6-Rb pathway with limited redundancies (Fig. 1a)^19^. Single genes encode for a *C. elegans* CDK4/6 kinase CDK-4, a D-type cyclin CYD-1 and a member of the Rb protein family, LIN-35. Candidate null mutations in *cyd-1* or *cdk-4* result in a general arrest of cell division in the G1 phase during larval development, slow growth and complete sterility (Fig. 1b)^20^. Inactivation of *lin-35* Rb by RNA interference (RNAi) or putative null mutation (*n745* and *n2239* alleles) suppresses the *cdk-4* CDK4/6 and *cyd-1* cyclin D mutant phenotype in part. Although *lin-35* Rb loss allows post-embryonic cell division in *cyd-1* and *cdk-4* mutants, double mutant animals that lack *lin-35* and *cyd-1*, or *lin-35* and *cdk-4*, remain small and sterile (Fig. 1b)^20^. In addition, quantification of cell numbers in the intestine and ventral nerve cord showed that the division of post-embryonic precursor cells remains limited in such double mutants (Fig. 1c,d)^20^,^21^. These data indicate that the CDK-4/CYD-1 kinase promotes cell cycle entry not only by inhibiting LIN-35 Rb but also through additional critical activities.

Additional functions could involve phosphorylation of other substrates or, as has been suggested for mammalian CDK4/6-cyclin D complexes^2^, sequestration of CDK-inhibitory proteins (CKIs; Fig. 1a). To examine whether the additional function of CDK-4/CYD-1 requires kinase activity, we expressed a FLAG-tagged kinase-dead (KD) form of CDK-4 in *lin-35*, *cdk-4* double-null mutants. As a control, we expressed wild-type (WT) *cdk-4::flag* introduced as a single-copy integrated transgene. Transgene-expressed WT *cdk-4::flag* completely rescued the *cdk-4(gv3)*-null mutant phenotype (Supplementary Fig. 1). Expression of KD CDK-4^D128E^::FLAG, however, neither alleviated the *cdk-4*-null phenotype nor improved rescue when combined with *lin-35* Rb inactivation (Supplementary Fig. 1). These data demonstrate that CDK-4/CYD-1 has one or more critical phosphorylation targets in addition to LIN-35 Rb.

### LIN-35 Rb and FZR-1 loss eliminates CDK-4/CYD-1 requirement

To identify critical substrates, we performed a genetic screen for mutations that suppress the cell division arrest and sterility of *lin-35*, *cyd-1* double mutant animals. In a large-size screen (>10,000 haploid genomes), we identified only a single mutant with complete rescue of the *lin-35(n745);cyd-1(he112)* phenotype. The mutant animals with this *he121* suppressor mutation appeared normal and produced progeny numbers similar to *lin-35* single mutants (Fig. 1b). Quantification of cell numbers further supported that postembryonic cell divisions in *cyd-1(he112)* mutants were fully restored by simultaneous *lin-35* inactivation and *he121* mutation (Fig. 1c,d). In fact, the triple-mutant larvae showed slightly higher than normal numbers of intestinal nuclei, again resembling the *lin-35* single-mutant phenotype (Fig. 1c, note: the normal intestine never has more than 34 nuclei). Combining *lin-35* RNAi with the *he121* mutation also restored cell division and fertility in *cdk-4(gv3)*-null mutants (Fig. 1b). Thus, rescue of the cell cycle arrest phenotype does not result from allele-specific suppression of *cyd-1(he112)*. Instead, the combined *lin-35* and *he121* mutations appear to abolish *cdk-4/cyd-1* requirement. Interestingly, the homozygous *he121* mutation also caused some suppression of the cell division arrest of *cyd-1* mutants with normal *lin-35* Rb function (Fig. 1c,d; note: *cyd-1(he112)* larvae always have 16 intestinal nuclei). These data indicate that the *he121* mutation disrupts a negative regulator of cell cycle entry, which acts in parallel to *lin-35* Rb.

We mapped the *he121* mutation to chromosome 2, to the right of *dpy-10* and close to map position 1. Whole-genome sequencing of the *lin-35(n745);he121 cyd-1(he112)* strain revealed a nonsense mutation in the fourth codon of the *fzr-1* FZR1 predicted open reading frame (CAT to TAG; Glu to Stop). *C. elegans fzr-1* encodes a co-activator and substrate specificity factor of the anaphase-promoting complex (APC/C) that is closely related to human FZR1 (also known as Cdh1), *Drosophila fizzy related (fzr)* and budding yeast Cdh1/Hct1 (refs 22, 23, 24). The APC/C^FZR1^ E3 ubiquitin ligase targets specific proteins for degradation in late mitosis and early G1. APC/C^FZR1^ is known to inhibit G1/S progression, which appears to agree with *he121* being an *fzr-1* allele. Additional observations support this conclusion: fosmid and plasmid clones carrying genomic *fzr-1* sequences complemented the *he121* phenotype when introduced as transgenes (Supplementary Table 1). Further, RNAi of *fzr-1*, obtained by soaking first stage (L1) larvae, rescued the cell cycle defects in *lin-35*, *cyd-1* double mutants and allowed production of viable offspring (Supplementary Table 2). However, *fzr-1* RNAi and the *fzr-1(ku298)* strong loss-of-function mutation predominantly induced lethality and sterility when combined with *lin-35* loss of function, as previously reported^24^. In combination with *cyd-1* loss, or double *lin-35*, *cyd-1* mutations, strong *fzr-1* RNAi also resulted in a higher number of nuclear divisions in the intestine, compared with the *fzr-1(he121)* mutation (Fig. 1c, note: the *P*-derived neuroblasts examined in Fig. 1d are somewhat RNAi insensitive). This combination of results indicates that *he121* is a partial loss-of-function allele of *fzr-1*. The effect of the *he121* early termination codon may be weakened by ribosomal read-through, translation initiation at a downstream ATG, or alternative usage of promoter sequences and splice sites. The requirement for an *fzr-1* allele to simultaneously suppress *cyd-1* loss and avoid synthetic lethality with *lin-35* may explain the identification of a rare partial loss-of-function *fzr-1* mutation in a large-size screen.

### Arrest of cell cycle entry by FZR-1 and LIN-35 Rb

**Figure 2:**
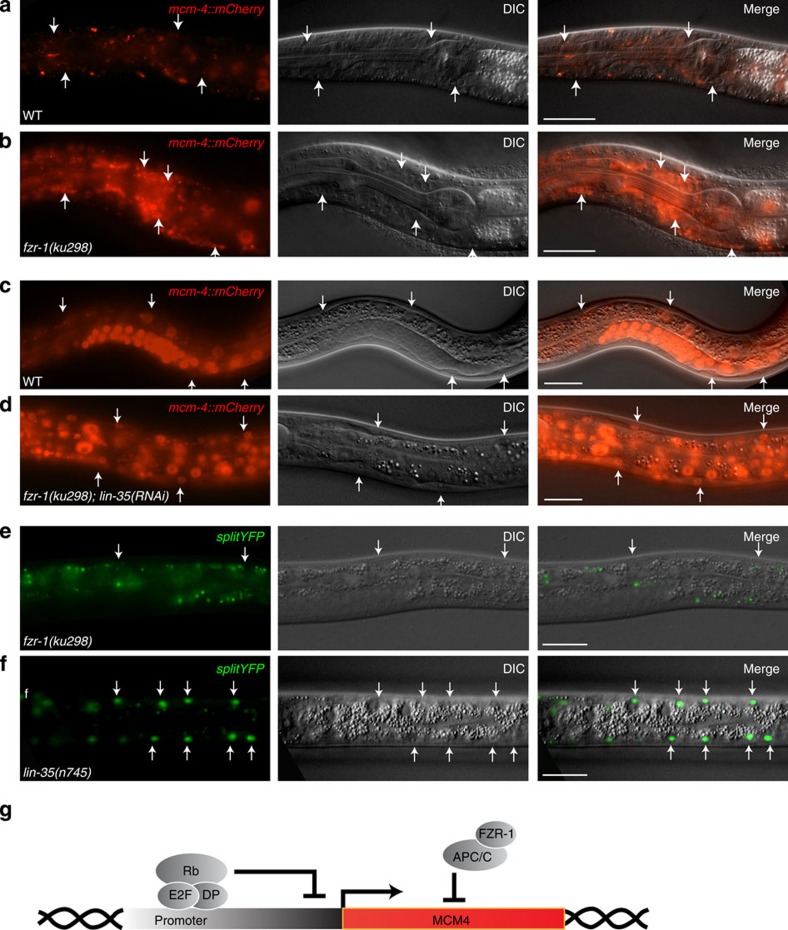
Differentiated cells in *lin-35; fzr-1* double mutants express S phase reporters. (**a**–**d**) Animals containing a translational S phase reporter of genomic *mcm-4* sequences, encoding an MCM-4 DNA replication helicase subunit fused to mCherry. (**a**) WT animal, head region. (**b**) *fzr-1(ku298*) mutant, head region. Note MCM-4::mCherry protein expression in many neurons of the nerve ring around the pharynx. (**c**) WT animal and (**d**) *fzr-1(ku298); lin-35(RNAi)* mutant. Note MCM-4::mCherry protein expression in body wall muscle cells (bwm), exclusively in the double mutant. (**e**,**f**) Animals containing a ‘split YFP’ transcriptional S phase reporter, with the N-terminal half of YFP expressed under control of the *mcm-4* G1/S-induced promoter, and C-terminal YFP expressed from the muscle specific *myo-3* promoter. (**e**) *fzr-1(ku298)* and (**f**) *lin-35(n745)* mutant. Note YFP expression in post-mitotic muscle cells, only in *lin-35(n745)* mutants. (**g**) Model illustrating parallel functions in cell cycle arrest through transcriptional repression by LIN-35 Rb and protein degradation by APC/C^FZR-1^. Arrows indicate individual cells that either do not express (**a**,**c**,**e**) or express (**b**,**d**,**f**) the reporter. Experiments were repeated four (**d**) or more times with many animals. Scale bars, 20 μm.

To examine cooperation between *fzr-1* and *lin-35* in cell cycle arrest, we tested how inactivation of these genes affects expression of *mcm-4* S phase reporter constructs. MCM-4 is a subunit of the replicative helicase complex, which we previously observed to accumulate in G1/S, and to be induced by cyclin D and cyclin E CDK activity^25^,^26^. We used a *Pmcm-4::MCM-4::mCherry* reporter to detect *mcm-4* transcription and protein expression. Remarkably, loss of *fzr-1* function by RNAi or use of the *ku298* allele resulted in MCM-4::mCherry protein expression in post-mitotic neuronal cells (Fig. 2a,b). This indicates that the strict G0/G1 phase arrest of differentiated neurons involves *fzr-1* function (Fig. 2b). Neurons did not initiate DNA synthesis in *fzr-1* mutants, based on the absence of EdU (5-ethynyl-2′-deoxyuridine) incorporation, and MCM-4::mCherry was not detected in other differentiated cells, such as muscle cells (Supplementary Fig. 2). *lin-35* Rb mutants did not display deregulated expression of MCM-4::mCherry in any cell type (Table 1). To specifically examine loss of transcription regulation, we designed a split-yellow fluorescent protein (YFP) reporter combination for detection of S phase gene activation in differentiated muscle cells. We combined expression of an N-terminal YFP fragment (*Pmcm-4::nzYFP*) under the control of a cell cycle promoter, with expression of a carboxy-terminal YFP fragment from a muscle-specific promoter (*Pmyo-3::czYFP*). In contrast to WT or *fzr-1* mutant animals (Fig. 2e), muscle-specific YFP fluorescence was observed in *lin-35* mutants, indicating that the *mcm-4* promoter is activated or de-repressed in the mutant muscle cells (Fig. 2f). Combining *lin-35* Rb and *fzr-1* mutations in *Pmcm-4::MCM-4::mCherry* transgenic animals caused broad expression of the MCM-4::mCherry fusion protein, including body wall muscle expression (MCM-4::mCherry expression in bwm of 152/186 animals; Fig. 2d and Table 1). Thus, LIN-35 Rb-mediated transcriptional repression and APC/C^FZR1^-mediated protein degradation act in parallel to prevent S phase gene expression, not only in cells that are temporarily quiescent but also in differentiated postmitotic cells (Fig. 2g).

### Posttranslational regulation of FZR-1 and LIN-35 function

**Figure 3:**
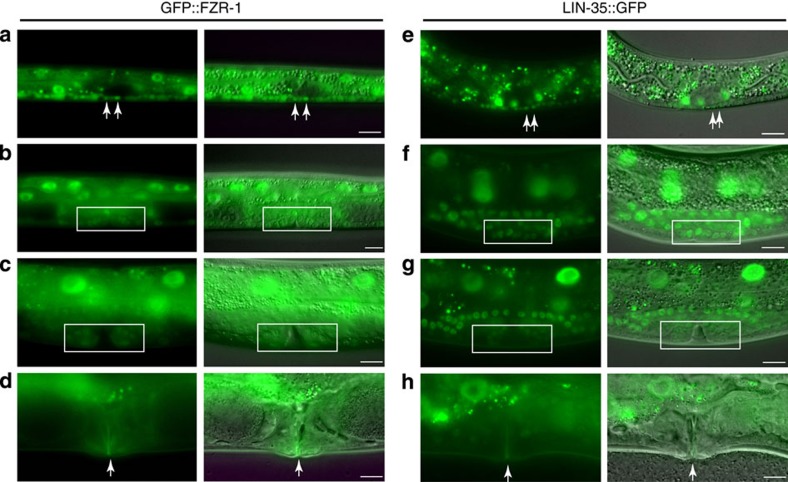
Expression of GFP-tagged FZR-1 and LIN-35 Rb correlates with cell cycle progression. (**a**–**h**) Fluorescence microscopy images of GFP expression and corresponding overlays of fluorescence and DIC images. (**a**,**e**) First-stage (L1) larvae showing GFP::FZR-1 (**a**) and LIN-35::GFP (**e**) expression. Arrows indicate dividing P cells with high expression levels. Dark spots in the ventral nerve cord are post-mitotic juvenile neurons. (**b**–**d** and **f**–**h**) Vulval development during the L3 stage. The vulval precursor cells (VPCs) close to the gonad migrate and divide to form the vulva (white boxes). The dividing VPCs express GFP::FZR-1 (box in **b**,**c**) and LIN-35::GFP (box in **f**,**g**). On completion of vulval formation, expression of FZR-1::GFP and LIN-35::GFP disappears from vulval cells (arrows in **d** and **h**). Images illustrate observations of many animals on multiple (>4) days. Scale bars, 10 μm.

We wondered whether the FZR-1 protein levels or localization are cell cycle regulated and reflect cell cycle stage-specific functions. We created transgenic animals that express FZR-1 with an N-terminal green fluorescent protein (GFP) tag, to be able to follow the FZR-1 protein throughout development in living animals. The GFP::FZR-1 fusion protein is functional, as it rescues the lethal phenotype of *lin-35(n745);fzr-1(ku298)* double mutants. Transgenic animals showed expression of GFP::FZR-1 in many somatic cells during larval development, at levels that varied in a cell type- and developmental stage-dependent manner (Fig. 3, left panels). In developmentally arrested L1 larvae, GFP::FZR-1 expression was detectable in the cytoplasm and nucleus of postembryonic precursor cells, with the highest levels in nuclei of the syncytial epidermis (hyp7) and intestine, and lowest levels in epidermal seam cells (Fig. 3a and Supplementary Fig. 3). Initiation of cell cycle entry during larval development coincided with a substantial increase in nuclear GFP::FZR-1 (observed in ventral cord precursor (P) cells, vulval precursor cells, intestinal cells and seam cells; Fig. 3a–c). Subsequent phases of temporal or permanent cell cycle arrest corresponded to a gradually declining GFP::FZR-1 signal (Fig. 3d, vulval precursor cells). Similarly, cells in *cyd-1* mutants that fail to enter S phase showed reduced GFP::FZR-1 expression (Supplementary Fig. 3). Hence, GFP::FZR-1 levels are high in actively cycling cells and decrease in cells arrested in G0/G1. It is surprising that GFP::FZR-1 levels are relatively low in G0/G1, when APC/C^FZR-1^ is active. This could possibly result from FZR-1 acting as an activator as well as a substrate of the APC/C E3 ubiquitin ligase. Alternatively or in addition, FZR-1 and other negative cell cycle regulators may be transcriptionally induced during cell division, to allow immediate arrest when needed. Notably, expression of LIN-35::GFP also correlated with cell division and decreased after cell cycle exit (Fig. 3, right panels). The abundance of LIN-35 and FZR-1 in dividing cells indicates that the activity of these negative regulators of cell cycle progression is predominantly controlled at levels other than protein expression.

### LIN-35 Rb and FZR-1 are CDK-4/CYD-1 kinase substrates

**Figure 4:**
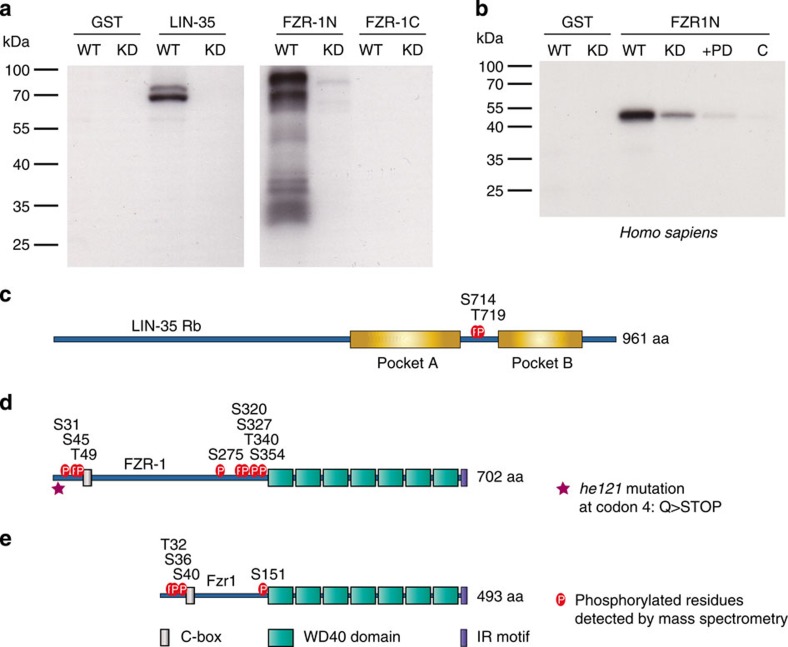
Phosphorylation of FZR1 by the CDK4-cyclin D kinase. (**a**) Autoradiogram following SDS–PAGE of *in vitro* kinase assays. FLAG-tagged *C. elegans* CDK-4 (WT) or KD CDK-4 (KD) were immunopurified and incubated with ^32^P-γ-ATP, and the purified GST protein alone, or GST-fused to either the LIN-35 pocket region, FZR-1 N terminus or FZR-1 C terminus. (**b**) Same as in **a**, but kinase assays were performed with FLAG-tagged human CDK4 (WT) or KD CDK4 (KD), in the presence of GST alone or GST-fused to the human FZR1 N terminus. The KD control showed some kinase signal in multiple experiments, probably through contaminating kinases. In the +PD lane, 0.9 μM of the CDK4/6 inhibitor PD-0332991 was added to the reaction with CDK4 (WT). Last lane (C) shows a negative control kinase assay, using anti-FLAG IP from cells transfected with empty vector (**c**). Illustration of the *C. elegans* LIN-35 protein showing the location of the two CDK-4 phosphorylated residues (P) in the spacer region between the pocket A and B domains. (**d**) Illustration of the *C. elegans* FZR-1 protein, indicating the positions of CDK-4 phosphorylated residues (P) and protein domains. The asterisk marks the *he121* mutation. The C-box is located at a.a. 59–65, the 7 WD40 domains start at a.a. 388, the final two a.a. form the IR motif. (**e**) Illustration of the *H. sapiens* FZR1 protein, the positions of the CDK-4 phosphorylated residues (P) and protein domains are indicated. C- box: a.a. 43–55, the 7 WD40 domains start at a.a. 182, the final two a.a. form the IR motif. All kinase assays and mass spectrometric analysis of the *C. elegans* proteins were performed twice, mass spectrometry of human FZR1 once.

Our genetic observations demonstrate that the CDK-4/CYD-1 kinase is required for cell cycle entry, unless *lin-35* Rb and *fzr-1* are simultaneously inhibited. The simplest explanation for these findings is that LIN-35 and FZR-1 are the main targets of CDK-4/CYD-1, and that phosphorylation inhibits LIN-35 and FZR-1 function. Similar to Rb, FZR1 (Cdh1) activity is known to be temporally regulated by CDK activity^27^,^28^,^29^. Activation of APC/C^FZR1^ follows mitotic cyclin destruction and CDK1 inactivation in anaphase. In the next cell cycle, phosphorylation of FZR1 at the G1/S transition and association with the Emi1 pseudo-substrate inhibitor are thought to switch off APC/C^FZR1^ activity^29^,^30^. Although in particular CDK2-cyclin A has been implicated in this phosphorylation^31^, it appears likely to be that CDK4/6-cyclin D contributes to overcoming APC/C^FZR1^-mediated G1 inhibition when cell cycle entry depends on this kinase. To test this possibility, we performed kinase assays *in vitro*, thereby avoiding indirect phosphorylation events by other CDKs and making use of the well-established substrate specificity of CDK4/6-cyclin D kinases *in vitro*. In these assays, we used *C. elegans* CDK-4/CYD-1 expressed and immunopurified from HEK 293T cells in combination with the bacterially expressed substrates GST-LIN-35 and GST-FZR-1. Autoradiography and mass spectrometric analysis revealed efficient and specific phosphorylation of both substrates (Fig. 4a). LIN-35 Rb was phosphorylated by CDK-4/CYD-1 at Ser714 and Thr719, located between the pocket A and B regions (Fig. 4c). These phosphorylations resemble Ser608 and Ser612 phosphorylation of human Rb, which reduce the affinity for E2F by promoting self-association between the Rb pocket loop and E2F-binding site of Rb^32^. Although human CDK4/6-cyclin D can phosphorylate Ser608 and Ser612, the specificity of this phosphorylation remains disputed^33^.

Mass spectrometric analysis of *in vitro* phosphorylated FZR-1 revealed eight CDK-4/CYD-1-phosphorylated residues in the N-terminal half of FZR-1 (Fig. 4d). Phosphopeptides containing these residues were abundant in the WT CDK-4 kinase reactions and were either not detectable or present at very low levels in the kinase dead CDK-4 control (Supplementary Table 3). All eight phosphorylated residues are followed by a proline residue and four are part of a T/SPxR/K consensus CDK phosphorylation site. For comparison, we also expressed human CDK4-cyclin D2 and found efficient phosphorylation of a human FZR1 N-terminal fragment (Fig. 4b). Mass spectrometric analysis identified four residues that were differentially phosphorylated by CDK4-cyclin D2 compared with the KD CDK4 control (Fig. 4e). These four phosphorylated residues of human FZR1 are also part of proline-containing sites. Moreover, their location in the FZR1 N-terminal domain is similar to the location of the CDK-4/CYD-1-phosphorylated residues of *C. elegans* FZR-1 and at least two of the phospho sites are evolutionarily conserved (*C.e.* Ser45, Thr49 versus *H.s.* Ser36, Ser40).

A previous study showed that mutation to alanine of four conserved CDK consensus sites prevented inactivation of human FZR1 (Cdh1)^31^. Two of these sites, Ser40 and Ser151, were phosphorylated by CDK4 in our *in vitro* kinase assays. Moreover, previous quantitative phosphorylation analysis showed cell cycle-dependent *in vivo* phosphorylation of FZR1 at five N-terminal serine residues, including Ser32, 36 and 151 (ref. 34). Together, our data indicate evolutionarily conserved inhibitory phosphorylation of Rb and FZR1 proteins, and are consistent with LIN-35 and FZR-1 inactivation in G1 through phosphorylation by CDK-4/CYD-1.

### LIN-35 Rb and FZR-1 probably are CDK-4/CYD-1 targets *in vivo*

We considered alternative or complementary mechanisms for the elimination of CDK-4/CYD-1 requirement following Rb and FZR-1 reduction. Based on results in other systems, loss of FZR-1 might allow persistence of mitotic cyclins into the next G1 phase, or lead to premature CDK-2/CYE-1 cyclin E activation. The inappropriate activity of cyclin B or cyclin E kinases could bypass the CDK-4/CYD-1 kinase requirement. Immunohistochemical staining of *fzr-1(he121)* and *fzr-1(ku298)* mutants indicated normal degradation of CYB-1 cyclin B1 and CYB-3 cyclin B3 in mitosis (Supplementary Fig. 4a–c). This is in agreement with mitotic cyclin degradation by APC/C in association with the Cdc20/Fizzy substrate specificity factor. Immunohistochemical staining of CYE-1 cyclin E also did not reveal apparent abnormalities, as CYE-1 expression was only detected in cells expected to be in S phase. Western blotting experiments were not conclusive but at most indicated slightly increased CYE-1 levels in *fzr-1(ku298)* and *lin-35(n745);fzr-1(he121)* larvae (Supplementary Fig. 4). Human APC/C^FZR1^ regulates SCF^Skp2^ and thereby p21^Cip1^ and p27^Kip1^ degradation^35^,^36^. In analogy, activation of CDK-2/CYE-1 could also indirectly result from CKI-1 CIP/KIP depletion following *fzr-1* loss. However, CKI-1::GFP levels were increased, rather than decreased, following RNAi of *fzr-1*, indicating that this control is not conserved in *C. elegans* (Supplementary Fig. 5). Thus, although CDK-2/cyclin E may replace CDK-4/CYD-1 function, our data favour a model in which CDK-4/CYD-1 kinase activity is required to counter LIN-35 Rb- and APC/C^FZR1^-mediated G1 inhibition.

### CDK4/6 inhibitor-induced arrest of human cancer cells

**Figure 5:**
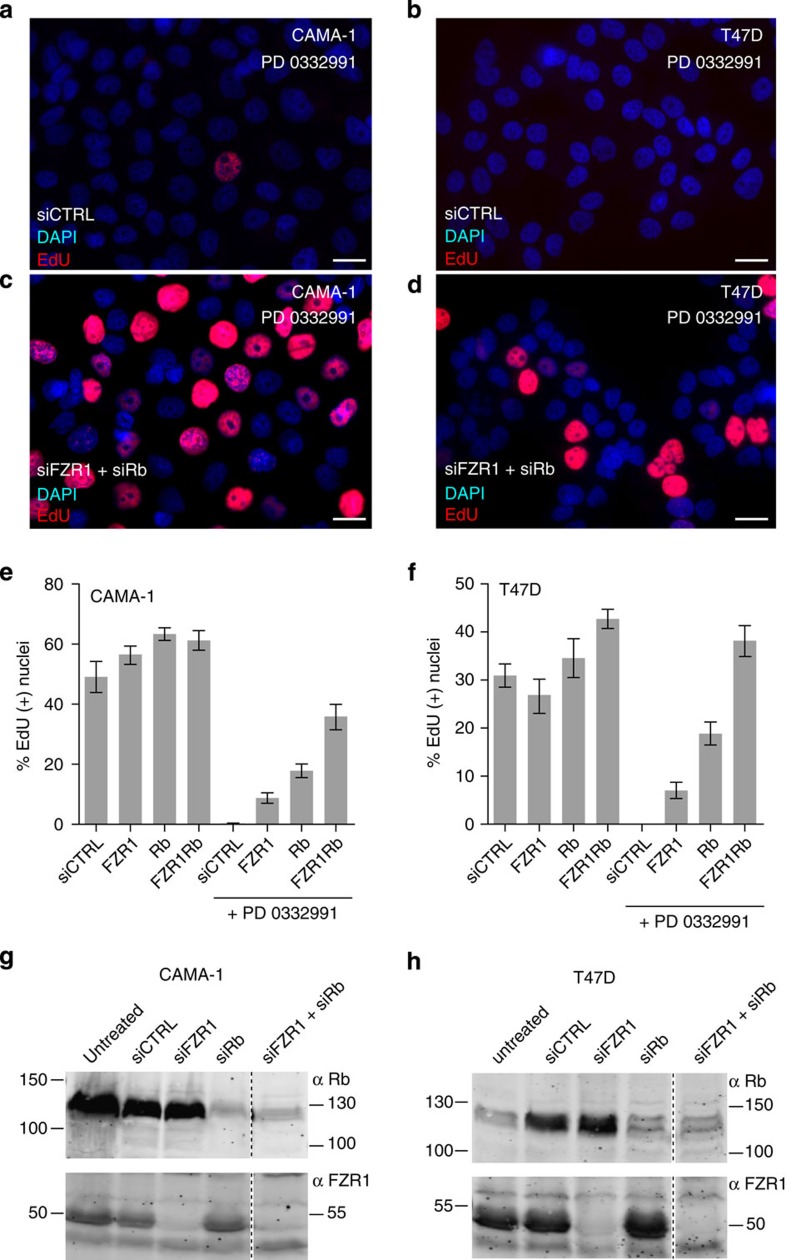
Knockdown of Rb and FZR1 overcomes cell cycle arrest induced by CDK4/6-inhibitor treatment of human breast cancer cell lines. (**a**–**d**) Representative immunofluorescence images of a field of CAMA-1 (**a**,**b**) and T47D (**c**,**d**) cells, following visualization of DNA replication by EdU incorporation and staining, and nuclei with DAPI staining. Cells were first transfected with nonspecific control siRNAs (siCTRL) (**a**,**c**), or combined FZR1- and Rb-specific siRNAs (**b**,**d**), treated with the CDK4/6 inhibitor PD-0332991 (500 nM) and subsequently incubated with EdU. Scale bar, 10 μm. (**e**,**f**) The percentage of EdU-positive nuclei counted for CAMA-1 (**e**) or T47D (**f**) cells treated with siRNA pools as indicated, in the absence or presence of PD-0332991 (500 nM). (**g**–**h**) Western blottings of CAMA-1 (**g**) or T47D (**h**) total cell lysates, following transfection with the indicated siRNA pools. Blots were probed with either anti-Rb or anti-FZR1 antibodies. All aspects of the experiments were performed twice, with similar results.

Given our results in *C. elegans*, we wondered whether human tumour cells require CDK4/6-cyclin D activity to counteract APC/C^FZR1^. To test this possibility, we combined treatment of human breast cancer cells with the CDK4/6-specific inhibitor PD-0332991 with knockdown of Rb and FZR1. As expected, treatment with 500 nM PD-0332991 induced cell division arrest in CAMA-1 luminal-type breast cancer cells and T47D ductal mammary carcinoma cells^15^. We examined incorporation of the thymidine analog EdU, which confirmed that the arrested cells lack DNA synthesis, and thus S phase (Fig. 5a,b). Transfection of CAMA-1 and T47D cells with pools of small interfering RNAs (siRNAs) targeting Rb or FZR1 messenger RNA caused substantial downregulation of the corresponding proteins (Fig. 5g,h). FZR1 knockdown resulted in limited rescue of the cell cycle arrest induced by PD-0332991 in CAMA-1 and T47D cells (Fig. 5e,f). The effect of Rb knockdown was somewhat stronger, but still limited. In contrast, double inhibition of FZR1 and Rb strongly rescued the cell cycle arrest induced by PD-0332991 in both cell types (Fig. 5c–f). These synergistic interactions between Rb and FZR1 closely resemble the observed interactions between *cdk-4* CDK4/6, *cyd-1* cyclin D, *lin-35* Rb and *fzr-1* FZR1 in *C. elegans* (Fig. 1c,d). Together, our data indicate that not only the mutant or WT status of Rb but also the expression levels of FZR1 and functional activity of the APC/C^FZR1^ E3 ligase will determine how cancer cells respond to CDK4/6 inhibitor treatment.

## Discussion

We found that combined inhibition of the Rb transcriptional repressor and the FZR1 APC/C co-activator eliminates the normal requirement for CDK4/6-cyclin D kinase activity in *C. elegans* and human breast cancer cells. In addition, we showed that CDK4-cyclin D phosphorylates FZR1 and Rb at multiple target sites *in vitro*. These phosphorylated residues are present at very similar locations in the *C. elegans* and human Rb and FZR1 proteins. Previous studies have shown that phosphorylation at these sites corresponds to inhibition of human Rb or FZR1 (refs 31, 32). As CDK4/6-cyclin D kinases are notoriously selective even *in vitro*, these combined observations make FZR1 a candidate novel target for regulation by cyclin D kinase phosphorylation.

FZR1 is a well-known CDK substrate, but previous studies have largely focused on inactivating phosphorylations that occur following the G1/S transition, in particular phosphorylation by CDK2-cyclin A and mitotic CDK1 (refs 27, 28, 29, 31). Our findings imply that regulation of APC/C^FZR1^ by CDK4/6-cyclin D forms part of the molecular network controlling passage through the G1 restriction point. At least one study in yeast implicated G1-CDKs in the inactivation of APC^Cdh1^ (ref. 37). However, other experiments showed that degradation of at least some of the substrates of APC^Cdh1^ continues into S phase, and that S phase cyclins are required for full APC^Cdh1^ inactivation^38^. Based on these data and our own results, we hypothesize that the inactivating phosphorylation of FZR1 may follow a pattern similar to Rb phosphorylation. As such, initial cyclin D-kinase phosphorylation might weaken the G1 inhibitory function of APC/C^FZR1^. Combined with a simultaneous reduction in Rb-mediated transcriptional repression by CDK4/6-cyclin D phosphorylation, this may allow some induction of CDK2-cyclin E activity. Phosphorylation by CDK2-cyclin E fully inactivates Rb and possibly APC/C^FZR1^, triggering a positive feedback loop and commitment to S phase entry. Further experimentation will be needed to test this model, and to explain how association with pseudo-substrate inhibitors, which provide an alternative mechanism for APC/C^FZR1^ inactivation, contributes to this switch.

The observed contribution of FZR1 loss in eliminating CDK4/6-cyclin D requirement could involve a more indirect mechanism. Mutation of Cdh1/Hct1 in yeast or *fzr* in *Drosophila* allows persistence of mitotic cyclins beyond M phase^22^,^39^. This ectopic cyclin expression triggered S phase in pheromone-exposed yeast cells and *Drosophila* cells that undergo a final mitosis^22^,^40^. Our EdU incorporation experiments in *C. elegans* did not reveal ectopic S phases, even when *fzr-1* was strongly inactivated. Moreover, *lin-35(0); fzr-1(he121)* double mutants and *fzr-1* strong loss of function mutants showed normal cyclin B1 (CYB-1) and cyclin B3 (CYB-3) degradation in mitosis. Therefore, we do not expect that persistence of mitotic cyclins is responsible for the *fzr-1* phenotype. The APC/C in association with its other co-activator, FZY-1 (Cdc20), probably retains prolonged activity in the absence of FZR-1 and suffices for mitotic cyclin degradation, as has been observed in human cells^41^,^42^.

As an alternative possibility, premature activation of cyclin E-kinase activity could bypass cyclin D-kinase requirement in *lin-35* Rb, *fzr-1* double mutants. Although loss of Rb is likely to lead to de-repression of *cye-1* cyclin E transcription, reduced APC/C^FZR-1^ activity has been reported to alleviate CDK2-cyclin E kinase inhibition in human cells^35^,^36^. The latter effect involves the F-box factor SKP2, which acts as the substrate specificity factor of the E3 ubiquitin ligase SCF. SKP2 is one of the best-characterized targets for APC/C^FZR1^ in human cells. Hence, downregulation of APC/C^FZR1^ activity leads to increased SCF^SKP2^ function, and thereby to degradation of its substrates, the p21^Cip1^ and p27^Kip1^ CDK inhibitors^35^,^36^. The protein expression level of CKI-1, the *C. elegans* homologue of Cip1/Kip1, was increased in *fzr-1* mutants, rather than reduced, compared to WT. This indicates that the link between APC/C^FZR1^ and Cip1/Kip1 regulation is probably not conserved in *C. elegans*. Therefore, we favour the model that LIN-35 Rb and FZR-1 are the two critical targets for inhibitory phosphorylation by the CDK-4/CYD-1 kinase, which when removed allow cyclin D-kinase-independent cell cycle entry.

We cannot exclude that the above-mentioned indirect mechanisms for elimination of CDK4/6-cyclin D requirement contribute to the escape from PD-0332991-induced arrest of human cancer cells following Rb and FZR1 knockdown. Independent of the mechanism, however, it is important to realize that reduced FZR1 levels correspond to reduced sensitivity for CDK4/6-specific inhibitors. At least three different CDK4/6-specific inhibitors are in various phases of clinical trials with human cancer patients^16^,^17^,^18^. Given the promise of these inhibitors in cancer treatment, it will be important to identify which genetic makeup corresponds with sensitivity to, or allows escape from, drug treatment. Although mutations in FZR1 are not common, the level of FZR1 protein expression and functional activity can be compromised in human cancer^42^. Probably reflecting this reduced activity, overexpression of the APC/C^FZR1^ substrate SKP2 has been observed in a subset of human cancers with poor prognosis^43^,^44^. Future studies should determine whether expression levels of FZR1 or its proteolytic targets such as SKP2 provide prognostic markers for the response of cancer cells to CDK4/6 inhibitor treatment.

## Methods

### *C. elegans* culture

Strains used are described in the Supplementary Table 4. Strains were cultured on Nematode Growth Medium (NGM) plates seeded with *Escherichia coli* OP50 according to standard protocol. All experiments were conducted at 20 °C, unless indicated otherwise. Feeding RNAi was performed on NGM plates supplied with 50 μg ml^−1^ Ampicillin and 2 mM IPTG. Soaking RNAi was performed as previously described^45^, using synchronized L1 larvae obtained by hypochlorite treatment and hatching of eggs in M9 medium with 0.05% Tween-20. L1 larvae were soaked in 10 μl dsRNA solution for 23 h at 20 °C, transferred to NGM plates with OP50 and allowed to develop for the appropriate amount of time.

### Genetics and whole-genome sequencing

For mutagenesis, L4 animals of strain SV357, *lin-35(n745)/dpy-5(e61) unc-29(e91)* I*; rol-1(e91) cyd-1(he112)/mnC1[dpy-10(e128) unc-52(e444)]* II, were collected in 1-ml M9 medium with 0.05% Tween-20, followed by addition of 20 μl of ethyl methane sulfonate and incubation for 6 h at room temperature. The F2 progeny was examined for fertile Rol animals. For genetic mapping of *he121*, we used mutations with visible phenotypes and single-nucleotide polymorphisms in Hawaiian strain CB4856. These experiments placed *he121* to the right of *dpy-10(e128)* and left of *rol-1(e91)*, probably close to position 1 cM on chromosome II. We performed high-throughput next-generation sequencing (SOLid) of three different strains. One was the backcrossed *he121* mutation strain isolated in the screen (SV383), one strain contains the *he121* mutation and homozygous *cdk-4(gv3)*, but no longer carries *cyd-1(he112)* (SV789), and one strain (SV331) was a precursor of the strain used in the mutagenesis.

Whole-genome sequencing revealed three missense or nonsense mutations associated with *he121:* mutations in *cdc-14* (II, −1.07 cM), *sra-3* (II, 1.53 cM) and *fzr-1* (II, 1.62 cM). In contrast to *fzr-1*, soaking with dsRNA of *cdc-14*, *sra-3* or the combination did not suppress the *cyd-1(he112)* phenotype. Moreover, through recombination with a *dpy-10(e128) unc-4(e120)* chromosome, we were able to remove the *cdc-14* and *sra-3* mutations but not the *fzr-1* mutation. The resulting *dpy-10(e128) fzr-1(he121) rol-1(e91) cyd-1(he112)* animals (strains SV1303 and SV1304) remained fertile on *lin-35* RNAi plates.

### DNA plasmid construction and transgenics

For primers used, see Supplementary Table 5.

*cdk-4::flag and cdk-4kd::flag*. To create the *cdk-4wt::flag* and *cdk-4kd::flag* microparticle bombardment constructs, we cloned a genomic EcoRV fragment containing the entire *cdk-4a* coding region as well as 3,466 bp of upstream and 770 bp of downstream sequences into vector pBluescript SK(+). To add a flag tag, site-directed mutagenesis was used to create an MfeI site at the stop codon (changing CAAGTGA to CAATTGA). A linker containing the flag tag, created by annealing oligonucleotides FLAG tag linker F and FLAG tag linker R was inserted into this MfeI site. For the KD construct, QuickChange site-directed mutagenesis was used to create an Asp/Asn substitution at amino-acid position 187 using primers Asp187Asn F and Asp187Asn R. To generate the final microparticle bombardment constructs, both the WT and KD *cdk-4* constructs were cloned into the *unc-119(wt)* vector pDP#MM016b^46^. These constructs were subsequently transformed into *unc-119(ed3)* worms by microparticle bombardment^47^.

*Pmcm-4::mcm-4::mCherry::mcm-4UTR* was generated by cloning the *mcm-4::mCherry* insert^26^ into the MosScI vector pCFJ178—Ti10882 (IV), followed by single-copy integration into the genome^48^.

*mcm-4 and myo-3 split-YFP*. To create splitYFP reporters, we generated *myo-3::NLSegl-13::nzYFP* and *myo-3::NLSegl- 13::czYFP* constructs. The *myo-3* promoter was isolated from pPD93.97 (A. Fire) and cloned into the pCGS1 vector. The nzYFP and czYFP-coding sequences were isolated from T4#712 and T4#713, respectively, and cloned into the pCGS1-*Pmyo-3* vector. A *C. elegans egl-13* nuclear localization signal was placed in frame between promotor and splitYFP DNA sequences.

The *myo-3* promoter of *myo-3::NLSegl-13::nzYFP* and *myo-3::NLSegl-13::czYFP* constructs were replaced by the *mcm-4* promoter, which was PCR amplified with primers *mcm-4*prom F and *mcm-4*prom R. Upstream ATGs and part of the 5′-untranslated region (UTR) were removed from *mcm-4::NLSegl-13::nzYFP* and *mcm-4::NLSegl-13::czYFP*, to obtain higher transgene expression. To construct MosSCI vectors, the *myo-3::nzYFP* and *myo-3::czYFP* were cloned in the vectors pCFJ151-p5605 (II) and pCFJ178—Ti10882 (IV), respectively. *mcm-4::NLSegl-13::czYFP▵ATG* was cloned in the pCFJ178—Ti10882 (IV) backbone. Integrated transgenic lines were generated as described^48^.

*GFP::fzr-1*. Fosmid WRM0635dH03 (Sourse BioScience) was used as a PCR template for *fzr-1* genomic sequences. PCR fragments of 2.2 kb *fzr-1* promoter sequences, codon-optimized GFP^49^ (0.87 kb, a kind gift of the Hyman lab), *fzr-1*-coding genomic sequences (3.4 kb) and *fzr-1* 3′-UTR (0.95 kb) were ligated together and cloned into pBluescript. Transgenic lines were generated by microinjection with *myo-2::tdTomato* as a co-injection marker, and strains with a high percentage of transmission as detected by expression of *myo-2::TdTomato* were used for FZR-1 protein expression and localization studies.

In *fzr-1* rescue experiments, fosmid WRM0635dH03 was injected, together with *myo-2::GFP* in strain SV439. Two transgenic lines transmitting the extrachromosomal array were further examined: SV1258 (Line 1) and SV1259 (Line 2).

*lin-35::GFP*. Fosmid WRM063cF08 (Source Bioscience) containing 20 kb upstream and 9.5 kb downstream sequences surrounding *lin-35* was used to generate *lin-35::GFP* with a recombination-based approach described previously^50^. This homologous genetic engineering technique in bacteria allowed us to integrate the GFP tag into a large genomic region of almost 40 kb, probably containing all the native regulatory sequences required for proper *lin-35* expression.

*cki-1::GFP*. To generate *cki-1::gfp*, the GFP tag was integrated into fosmid WRM0611ch10 (Source Bioscience) by using the same recombination technique described above.

*Constructs for protein expression in human cells*. To express *C. elegans cdk-4::3xFLAG* and *cdk-4KD*^*D187N*^*::3xFLAG* in human cells, *cdk-4* was PCR amplified with primers: *cdk-4 Xho*I_5′ F and *cdk-4* _1026_*Hd*III R, and cloned into XhoI and HindIII sites of pBluescript. The *cdk-4_KD*^*D187N*^ mutation was generated by site-directed mutagenesis with *cdk-4KD* primer. The 3xFLAG fragment was PCR amplified from the p3xFLAG-myc-CMV-24 expression vector (Sigma) with the primers 3xFLAG_*Hd*III F and 3xFLAG_*Pst*I R, and cloned behind *cdk-4* and *cdk-4KD* in pBluescript. Both *cdk4* and *cdk-4KD* were cloned behind the cytomegalovirus (CMV) promoter of pEGFP-N1 (BD Biosciences, GenBank accession code U55762).

To express human CDK-4::3xFLAG and CDK-4KD^D158N^::3xFLAG, plasmids pCMV-Cdk-4-HA and pCMV-Cdk4NFG-HA (Addgene, van den Heuvel^51^) were digested with XhoI and BstXI. A double-stranded G-block oligonucleotide *H.s._cdk-4_FLAG* was ligated into the XhoI/BstXI site, resulting in an in-frame 3xFLAG-tagged construct.

To express *C. elegans* CYD-1 cyclin D in human cells, *cyd-1* complementary DNA was PCR amplified from pCGS*myo-3::cyd-1* with the following primers: *cyd-1*_1 *Eco*RI_F and *cyd-1*_1218 *Sal*I_R. The resulting PCR fragment was cloned behind the pCMV promoter of pEGFP N1.

*GST fusion proteins*. Fragments encoding the N or C terminus of *C. elegans* FZR-1 were PCR amplified from a *C. elegans* cDNA library with *fzr-1*_1 *Sal*I-F and *fzr-1*_1197 *Not*1-R for the N terminus and for the C terminus, *fzr-1*_988 *Eco*RI-F and *fzr-1*_2107 *Not*I-R. Subsequently, the PCR fragments were cloned into pGEX 4T-3. For GST-LIN-35, a *lin-35* fragment encoding the extended C-terminal region with the A and B pocket domains was amplified by PCR from a cDNA library with *lin-35*_1486 *Sma*I-F and *lin-35*_2883_*Not*IR, and cloned into pGEX 4T-2.

*Human GST-FZR1 proteins*. To generate human GST-FZR1 proteins, *fzr1* cDNA was PCR amplified from a *Homo sapiens* cDNA library with *fzr1_Hs*_*Eco*RI_1 F and *fzr1_Hs*_*Not*I_598 R, and cloned into the EcoRI/NotI sites of pGEX4T-3, resulting in an in-frame GST fusion protein. The constructs were introduced into *E. coli* BL21 and expression was induced with 2 mM IPTG for 3 h. The GST fusion proteins were purified by binding to Glutathione Sepharose beads (GE Healthcare).

### Immunostaining and antibodies

Immunohistochemical analyses and staining of DNA with propidium iodide or 4',6-diamidino-2-phenylindole (DAPI) were performed as previously described^52^. EdU labelling and staining were performed according to a protocol using the Click-IT EdU Alexa Fluor 594 kit (Life Technologies) as previously described^26^,^52^. Primary antibodies used for immunofluorescent staining are as follows: mouse anti-CYE-1 (1:150, 17C8, a kind gift of E. Kipreos), mouse anti-AJM-1 (1:20, MH27, Developmental Studies Hybridoma Bank), rabbit anti-CYB-3 (1:100)^45^, mouse anti-FZR-1 (1:100, DH01, Thermo Scientific), mouse anti-Rb (1: 1,000, 4H1, Cell Signaling Technology). For anti-FLAG immunopurifications M2 anti-FLAG beads (Sigma) were used. Western blotting was performed as previously described^45^, using the following antibodies: rabbit anti-FLAG (1:5,000, F7425, Sigma), rabbit anti-GFP (1:1,000, Invitrogen), mouse anti-Tubulin (1:2,000, T9026, Sigma), mouse anti-CYE-1 (1:2,000, 17C8, a kind gift of E. Kipreos), rabbit anti-CYB-3 (affinity purified 1:2,000)^45^. See Supplementary Fig. 6 for uncropped images of immunoblottings. Cell numbers were determined in synchronized animal populations, following fixation with Carnoy’s solution and staining of DNA with propidium iodide^52^. No statistical method was used to predetermine sample size; instead, total cell numbers were counted for the indicated tissues in at least ten animals for each genotype. Usually, all available mutant animals were quantified.

### Microscopy analysis

Microscopy was performed at 20 °C. Two microscope setups were used: a wide-field immunofluorescence microscope and a spinning disc confocal microscope. The wide-field fluorescence microscope is a Zeis Axioplan2 upright microscope equipped with a HAL 100 halogen visible light source controlled by an internal shutter, an HXP 120 C metal halide fluorescence light source controlled by a Uniblitz VMM-D1 shutter, a × 25/0.8 NA imm Corr objective with DIC slider, × 63 and × 100 Plan-APOCHROMAT 1.4 NA objectives with DIC sliders, a DIC polarizer and an Axiocam MRm CCD monochrome camera. The filter turret is equipped with the following Zeiss filter sets: filter set 34 for DAPI (excitation: 390/22 nm band pass, dichroic mirror: 420 nm, emission: 460/50 nm band pass), filter set 13 for GFP (excitation: 470/20 nm band pass, dichroic mirror: 495 nm, emission: 505–530 nm band pass), filter set 46 for YFP (excitation: 500/20 nm band pass, dichroic mirror: 515 nm, emission: 535/30 nm band pass), filter set 31 for mCherry (excitation: 565/30 nm band pass, dichroic mirror: 585 nm, emission: 620/60 nm band pass) and a DIC analyser cube. The microscope and camera are controlled by Zeiss Axiovision 4.x imaging software. Confocal images were obtained with a Zeiss LSM 700 confocal microscope.

### Immunoprecipitation and *in vitro* kinase assays

pCMV plasmids expressing *C. elegans* CDK-4::3xFLAG or CDK-4KD^D187N^::3xFLAG were co-transfected with a pCMV::CYD-1 expression vector in HEK 293T cells (PAA, Pasching, Austria). Cells were lysed and immunoprecipitated in CDK-4 buffer^53^ with M2 beads (Sigma). Kinase assays were performed as described by incubation of the immunoprecipitation beads for 30 min at 25 °C, with bacterially produced GST only or GST fused to the *C. elegans* FZR-1 N terminus (a.a. 1–406), the FZR-1 C terminus (a.a. 330–702) or the extended pocket region of LIN-35 (a.a. 496–961). The reaction was performed in kinase buffer containing 200 μM ATP, 50 mM HEPES at pH 7.5, 10 mM MgCl_2_, 1 mM EGTA, 2 mM dithiothreitol phosphatase inhibitors (Roche) and 20 μCi [γ-32 P]ATP for radioactive kinase assays. Reactions were terminated by the addition of SDS (4 × sample buffer)^54^. See Supplementary Fig. 6 for uncropped images of gels.

A similar experiment was performed with human CDK4 and KD CDK4^D158N^, co-transfected and expressed with cyclin D2. Kinase assays were performed with GST only or GST fused to the *H. sapiens* FZR1 N terminus (a.a. 1–199). The CDK4/6 inhibitor PD-0332991 was added to a final concentration of 0.9 μM.

### Mass spectrometry

The mass spectrometry proteomics data have been deposited to the ProteomeXchange Consortium^55^ via the PRIDE partner repository (see accession codes section). Gel lanes (WT and KD) were cut into different bands, which were treated with 6.5 mM dithiothreitol for 1 h at 60 °C for reduction and 54 mM iodoacetamide for 30 min for alkylation. The proteins were digested overnight with trypsin at 37 °C. The resulting peptides were extracted with 10% formic acid.

The samples were analysed using a Proxeon Easy-nLC 1000 (Thermo Scientific) connected to an QExactive mass spectrometer (Thermo Scientific). The injected samples were first trapped (Dr Maisch Reprosil C18, 3 μm, 2 cm, 100 μm) at a maximum pressure of 600 bar with solvent A (0.1% formic acid in water) before being separated on an analytical column (Agilent Poroshell EC-C18, 2.7 μm, 40 cm, 50 μm) at a stable temperature of 40 °C. Peptides were chromatographically separated by a 90-min gradient from 7% to 30% solvent B (0.1% formic acid in acetonitrile) at a flow rate of 100 nl min^−1^. The column eluent was directly introduced into the elctrospray source of the mass spectrometer. The electrospray voltage was set to 1.7 kV using a fused silica capillary (360 μm o.d., 20 μm i.d., 10 μm tip i.d., constructed in-house). The mass spectrometer was used in a data-dependent mode, which automatically switched between MS and MS/MS using a Top10 method (higher-energy collision dissociation fragmentation). Alternativley, samples were also analysed in an Agilent HPLC 1200 series (Agilent) connected to an LTQ Orbitrap Velos (Thermo Scientific) using a data-dependent decision tree as before^56^.

### Proteome data analysis

Raw files were processed using Proteome Discoverer 1.4 (version 1.4.1.14, Thermo Scientific). Mascot (Matrix Science, version 2.4) was used as a search engine against a combined database of the human and *E. coli* proteomes in which GST-Frz1 and GST-LIN-35 sequences were added. Carbamidomethylation (C) was set as a fixed modification, and oxidation (M) and phosphorylation (STY) were set as variable modifications. The PhosphoRS 3.0 node was used for the phosphosite localization. Trypsin was specified as enzyme and up to two missed cleavages were allowed. The following parameters were used: 50 p.p.m. for the precursor mass tolerance and 0.05 Da for the fragment ion tolerance (0.5 Da for ion trap data). Data filtering was performed using Percolator, resulting in 1% false discovery rate. Additional filters were applied as follows: search engine rank 1 peptides, minimum of 7 residues per peptide and Mascot ion score above 20. Extracted ion chromatograms were obtained for each phosphopeptide in both the KD and WT kinase (CDK4) experiments. The intensities of all the phosphopeptides containing the same phosphosite(s) were summed (for example, missed cleavages and methionine oxidation) to obtain a total signal in each experiment. The intensities for the different charge states of the same phosphopeptide (for example, 2+, 3+, 4+ and so on) were also summed. When multiple phosphopeptides were identified from the same chromatographic peak, only one value was used for the calculation. Normal peptides (that is, non-phosphopeptides) from FZR1 were used to normalize the data between the two samples.

### Cell culture and inhibitor assays

HEK293T cells were cultured in 50% DMEM/50% F10 with L-glutamine (Lonza) supplemented with 10% FCS and antibiotics. Cell transfections were performed with 25 kD linear polyethyleninmine (Polysciences)^57^. CAMA-1 and T47D cells were cultured in RPMI-1640 medium (Life Technololgies), supplemented with 10% FCS. Cells were seeded on coverslips and transfected for 3 days with 10 nM of ON-TARGET plus SMARTpool siRNA (Thermo Scientific) targeting FZR1 and/or RB1, using HiPerFect as previously described^57^, and compared with cells transfected with luciferase-targeting siRNA^58^. The CDK4/6 inhibitor PD 0332991 was added in a concentration of 500 nM in dimethyl sulfoxide for 24 h. The Click-iT EdU Imaging Kit (Life Technologies) assays were performed according to the manufacturer’s instructions. Briefly, cells transfected for 3days with siRNA were incubated 7 h with 20 mM EdU, fixed, stained using Click-iT EdU Alexa Fluor 594 Imaging Kit and counterstained with DAPI. For quantification, ten independent fields spanning duplicate experiments were imaged for Alexa Fluor 594 and DAPI fluorescence, and counted for EdU nuclear positivity relative to total number of nuclei per field.

## Author contributions

I.T. and S.R. contributed to the experimental design, performed most *C. elegans* experiments, analysed the data and participated in manuscript preparation. B.P.B. performed and analysed the experiments with breast cancer cells, with help from A.A. J.M. and A.C. performed the mass spectrometry experiments and data analysis, with help from A.J.R.H. H.X. identified the *he121* allele, J.K. participated in the genetic mapping, M.B.W.P. performed phenotypic characterizations, E.C. contributed whole-genome sequencing and data analysis. T.v.M. assisted with the kinase assays. M.B. participated in the *C. elegans* experiments and provided feedback on the manuscript. S.v.d.H. designed the experiments, assisted with data analysis and prepared the manuscript.

## Additional information

**How to cite this article:** The, I. *et al*. Rb and FZR1/Cdh1 determine CDK4/6-cyclin D requirement in *C. elegans* and human cancer cells. *Nat. Commun.* 6:5906 doi: 10.1038/ncomms6906 (2015).

**Accession codes:** The mass spectrometry data were deposited in ProteomeXchange with the accession code PXD001501.

## Supplementary Material

Supplementary Information Supplementary Figures 1-6 and Supplementary Tables 1-5

## Figures and Tables

**Table 1:** Quantification of the number of animals expressing the *mcm-4* translational or transcriptional S phase reporter in differentiated body wall muscle cells of wild type animals (WT), *fzr-1(ku298)* mutants, *lin-35(n745)* mutants or double mutants.

**Body wall muscle**	**WT**	***fzr-1***	***lin-35***	***lin-35; fzr-1***
MCM-4::mCherry protein expression	**−** (Many)	**−** (1/96)	**−** (1/82)	**+** (152/186)
*mcm-4* promoter activity	**−** (Many)	**−** (Many)	**+** (288/300)	**+** (ND)

ND, note defined.

−/+ indicates expression, between brackets: number of animals with reporter expression versus total number examined.
