# Supplementary material for: Rb and FZR1/Cdh1 determine CDK4/6-cyclin D requirement in C. elegans and human cancer cells
Source: Nat Commun. 2015 Jan 6;6:5906. doi: 10.1038/ncomms6906 (PMC4354291; doi:10.1038/ncomms6906)
Supplement: Supplementary Information — Supplementary Figures 1-6 and Supplementary Tables 1-5 [file ncomms6906-s1.pdf]

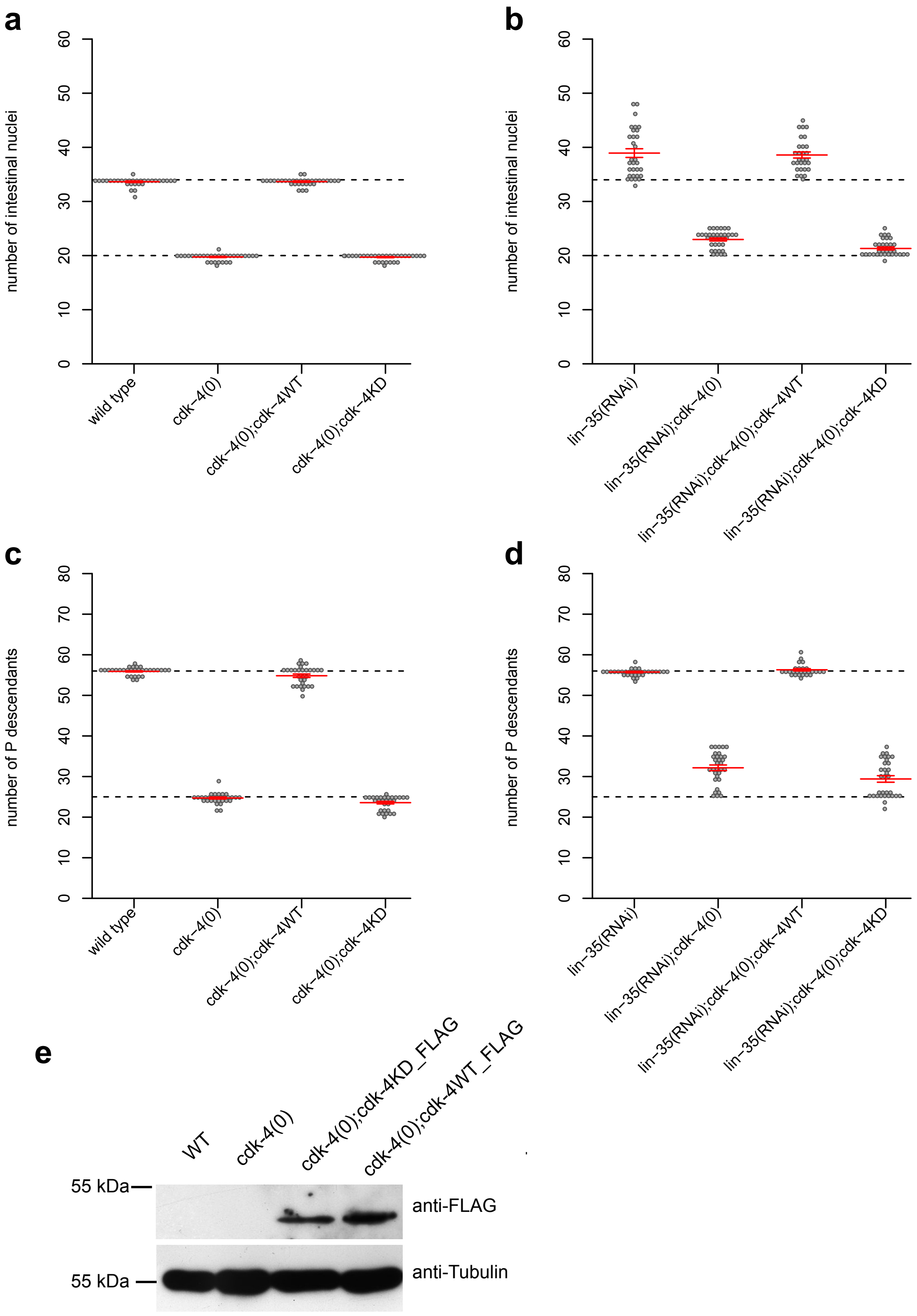

Supplementary Figure 1. Wild-type but not kinase-dead CDK-4 rescues cell division in *cdk-4* mutant animals. (**a**, **b**) Quantification of the number of intestinal nuclei for the indicated genotypes in the absence (**a**) or presence (**b**) of *lin-35* RNAi. (**c**, **d**) Quantification of the number of P cell descendants in the ventral nerve cord for the indicated genotypes, in the absence (**c**) or presence (**d**) of *lin-35* RNAi. (**e**) Western blot demonstrating expression of CDK-4WT-FLAG and CDK-4KD-FLAG. Equal amounts of total protein of the indicated strains were used for immunoprecipitation (IP) with anti-FLAG beads, followed by SDS-PAGE and immunobotting. Blots were probed with anti-FLAG antibodies to detect transgene expressed CDK-4. The anti-Tubulin blot illustrates equal amounts of protein used in the IP reactions. The graphs show mean ± s.e.m.; each dot represents a single animal. Total cell numbers were counted for the indicated tissues in at least 20 animals for each genotype.

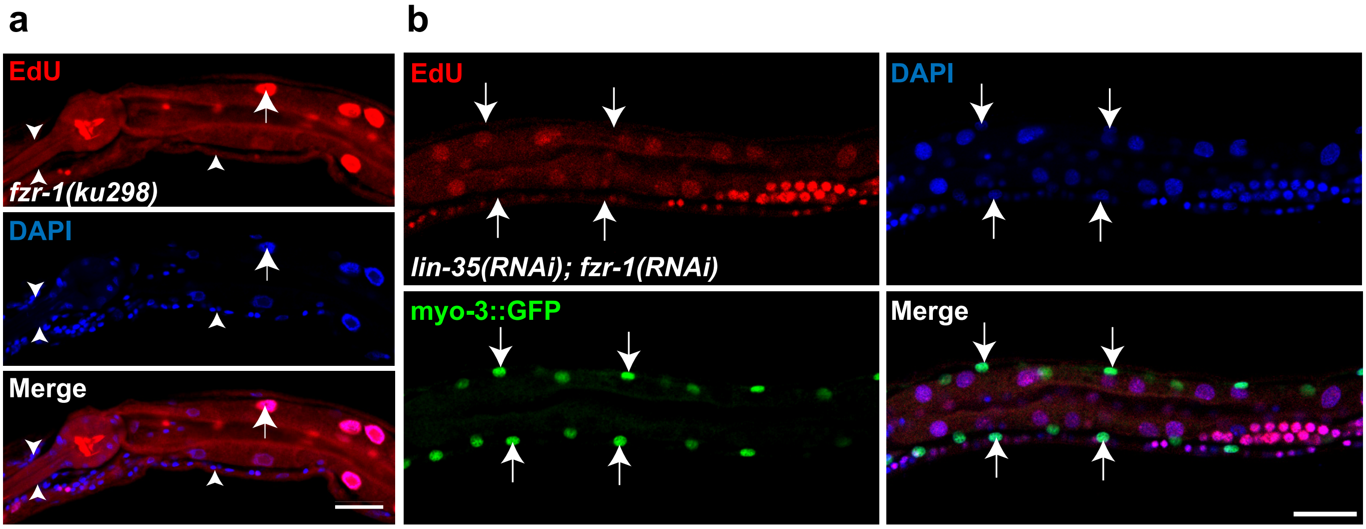

Supplementary Figure 2. Loss of *lin-35* and *fzr-1* does not lead to EdU incorporation in differentiated neurons and muscle cells. (**a**, **b**) Representative immunofluorescence images of EdU incorporation and staining experiments. (**a**) A *fzr-1(ku298)* mutant animal. EdU incorporation is seen in intestinal nuclei (arrow), which undergo endoreplication during larval development. Note that EdU is not incorporated in differentiated neurons in the head region and ventral nerve cord (arrowheads). (**b**) A *lin-35(RNAi); fzr-1(RNAi)* animal. Note that EdU incorporation is not detected in the body wall muscle cells (arrows) which are visualized by myo-3::GFP (green). All stained *fzr-1(ku298)* animals (many) were examined in three independent EdU labelling-staining experiments, staining of *lin-35(RNAi); fzr-1(RNAi)* larvae was performed once, with 28 animals analysed. Anterior is to the left in all panels. Scale bar: 20 μm.

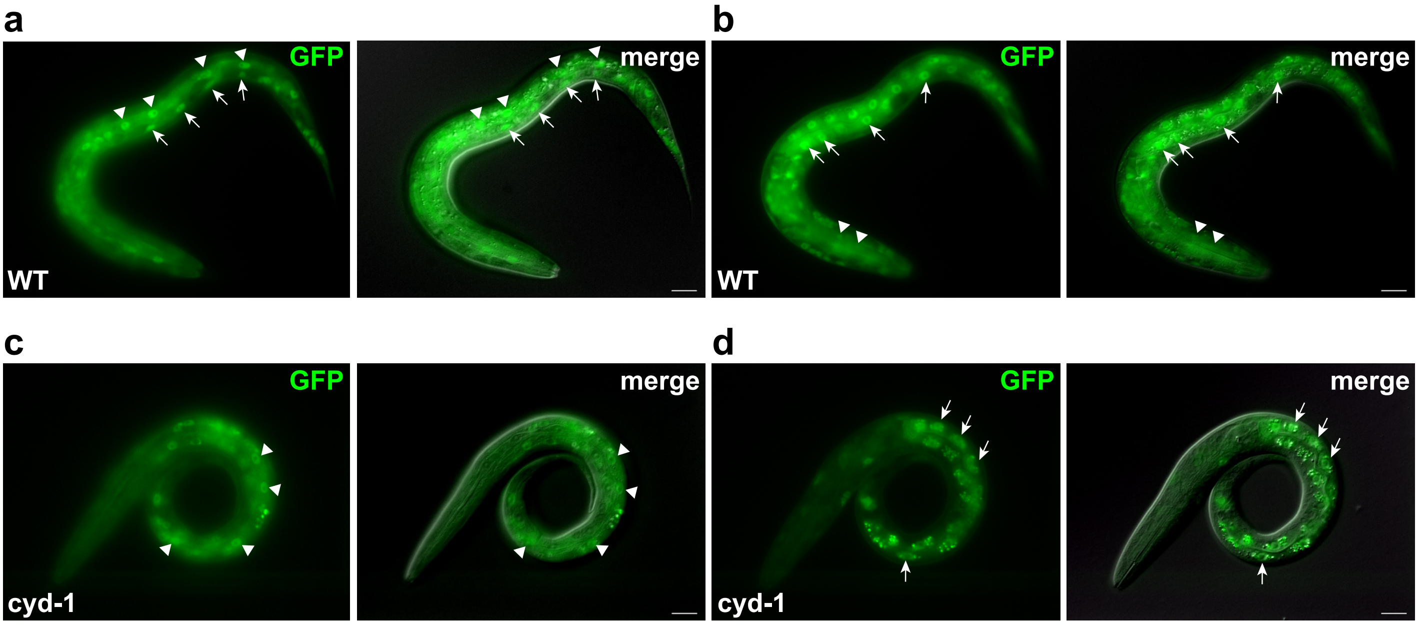

Supplementary Figure 3. GFP::FZR-1 expression in a wild type animal and *cyd-1* mutant larva. (**a**, **b**) In the starved L1 larva GFP::FZR-1 can be detected in the hypodermal nuclei (arrowheads in **a**), P cells (arrows in **a**), head neurons (arrowheads in **b**) and intestinal cells (arrows in **b**). (**c**, **d**) In *cyd-1* starved L1 larva GFP-FZR-1 is seen in the hypodermal cells (arrowheads in **c**) and intestinal cells (arrows in **d**). Dorsal is up in all pictures. The images represent observations of > 20 individual animals in >4 independent experiments for each genotype. Scale bar, 10 μm.

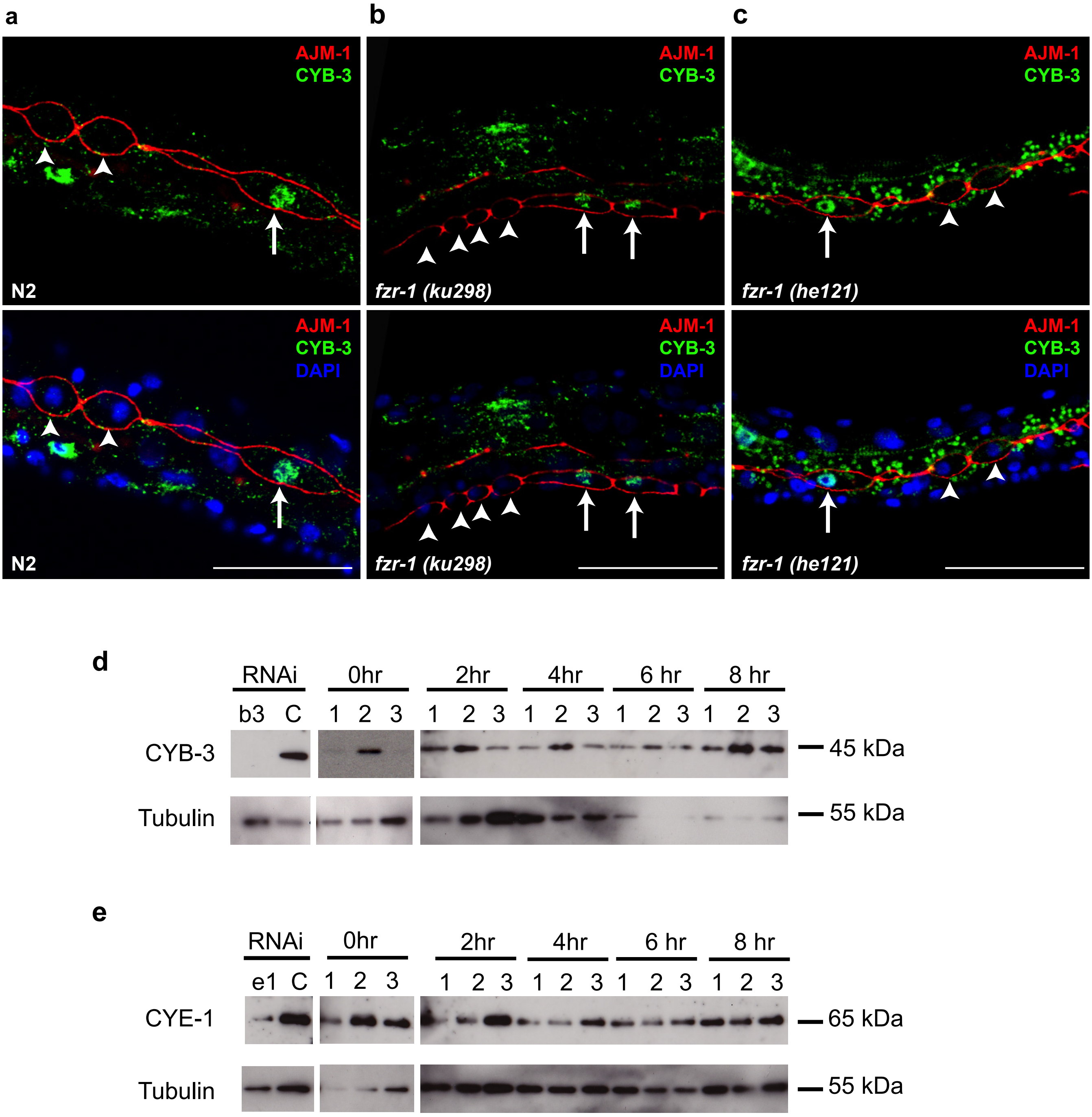

Supplementary Figure 4. Normal degradation of cyclin B3 (CYB-3) in *fzr-1* mutants. (**a-c**) Representative fluorescence microscopy images, following immunohistochemical analysis of CYB-3 expression in wild-type N2 (**a**), *fzr-1(ku298)* (**b**), and *fzr-1(he121)* (**c**) animals. Epithelial seam cell junctions are visualized with anti-AJM-1 staining, DNA with DAPI. Arrows indicates cells prior to division, in which CYB-3 levels are high. Arrowheads indicate cells that have just divided, in which expression of CYB-3 was not detected. (**d**, **e**): Western blot analysis of cyclin levels in *C. elegans* larvae from 0 to 8 hr of L1 development. (**d**) Upper panel anti-CYB-3 staining, lower panel anti-Tubulin. (**e**) Upper panel: anti-CYE-1, lower panel anti-Tubulin. As controls, *C. elegans* were fed with either *cyb-3* dsRNA (**d**: b3) or *cye-1* dsRNA (**e**: e1). Total embryo lysates of wild-type (N2, lanes 1), *fzr-1(ku298)* (lanes 2) or l*in-35(n745); fzr-1(he121) cyd-1(he112)* (lanes 3) mutants were immunoblotted and incubated with the indicated antibodies. Immunostaining and immuoblotting experiments were performed twice. All successfully stained animals with mitotic cells (n=5, n=8) were examined. Scale bar, 20 μm.

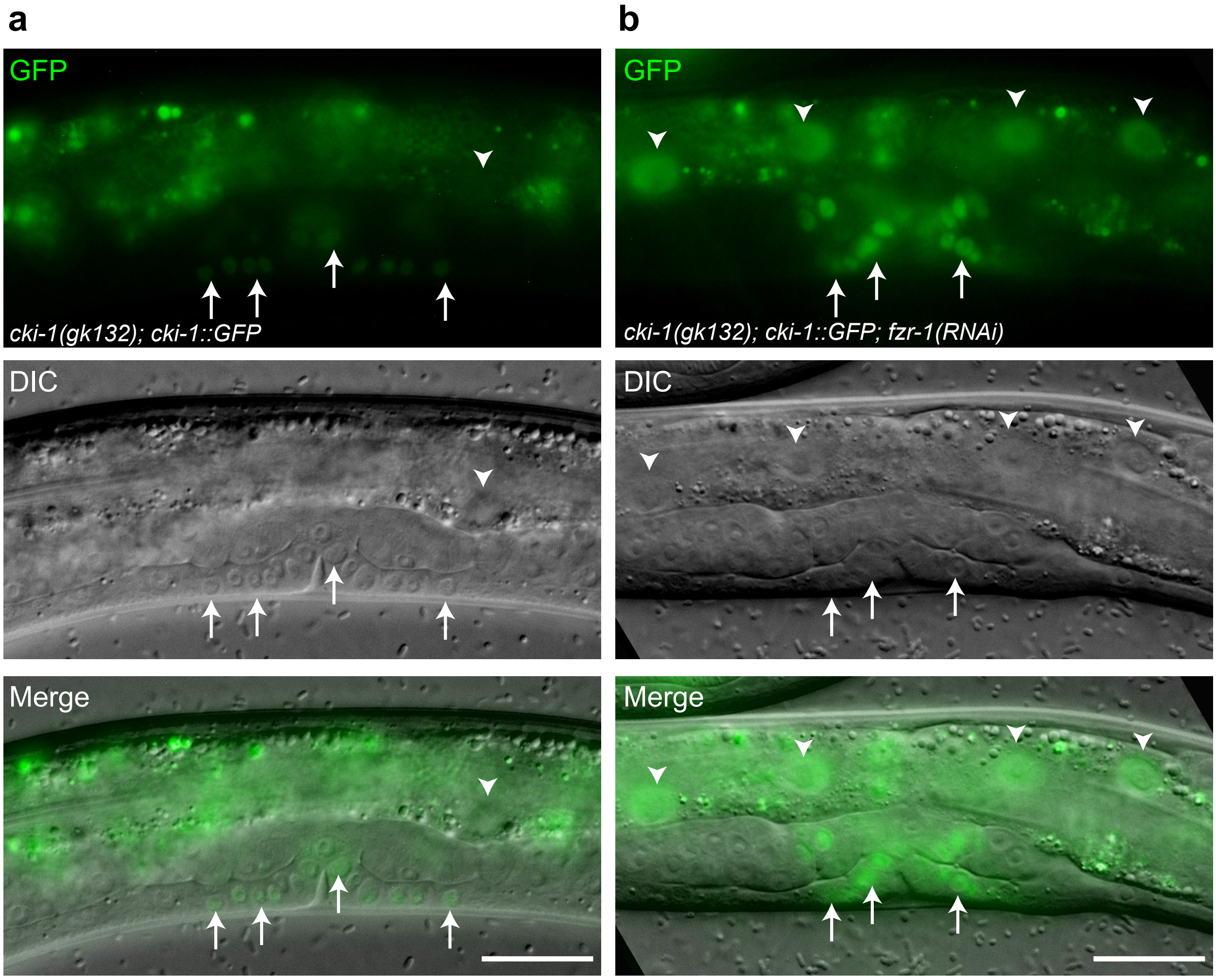

Supplementary Figure 5. CKI-1::GFP expression increases following *fzr-1* RNAi. (**a**, **b**) Representative fluorescence microscopy images of *cki-1(0)* L3 larvae expressing CKI-1::GFP from a fosmid-based transgene. The animal in (**b**) was treated with *fzr-1* RNAi. Note the increased CKI-1::GFP expression in the vulval cells (arrows) and intestinal nuclei (arrowheads). The experiment was repeated twice, each time > 20 animals were examined for each condition. Scale bar, 20 µm.

(gels of Figure 4)

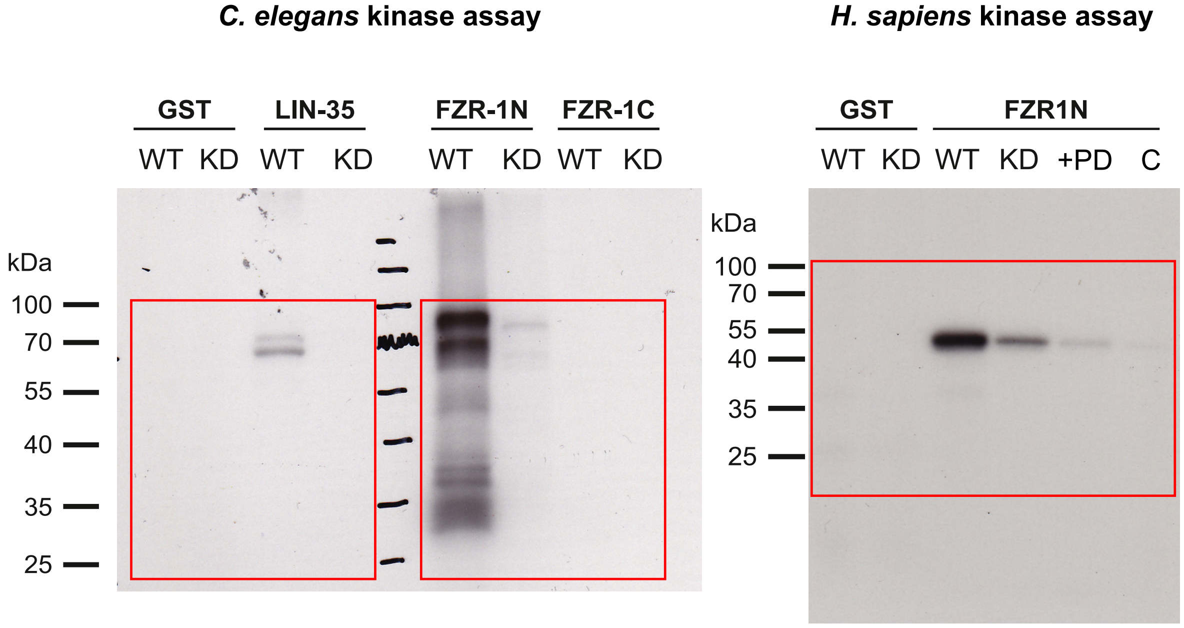

(blots of Figure 5)

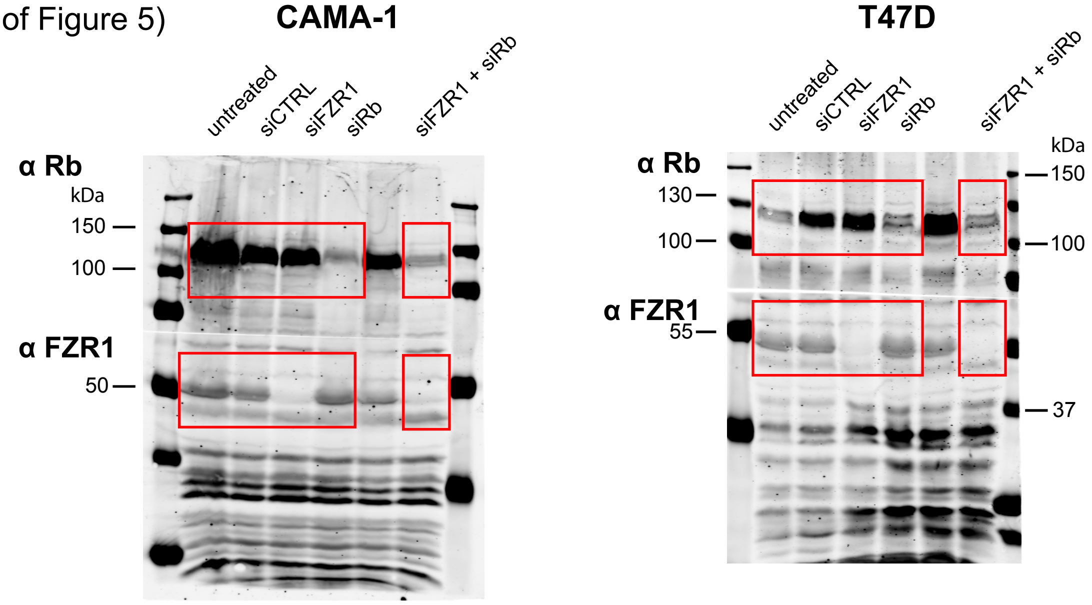

(blots of Supplementary Figure 1)

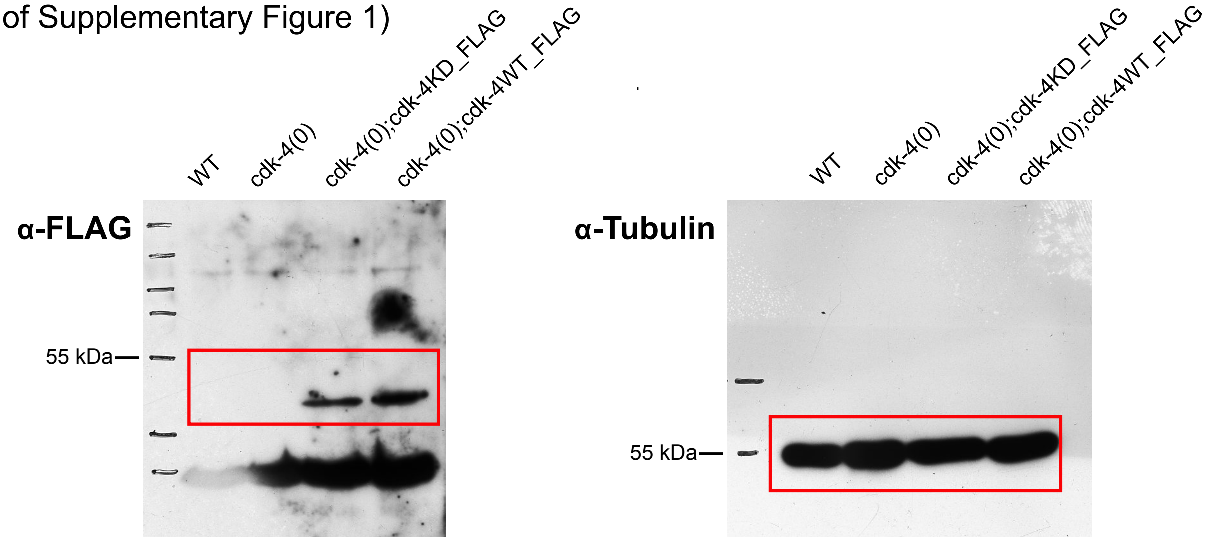

Supplementary Figure 6. Uncropped scans of gels and immunoblots. Boxes highlight the area selection used in the indicated figures.

(blots of Supplementary Figure 4d)

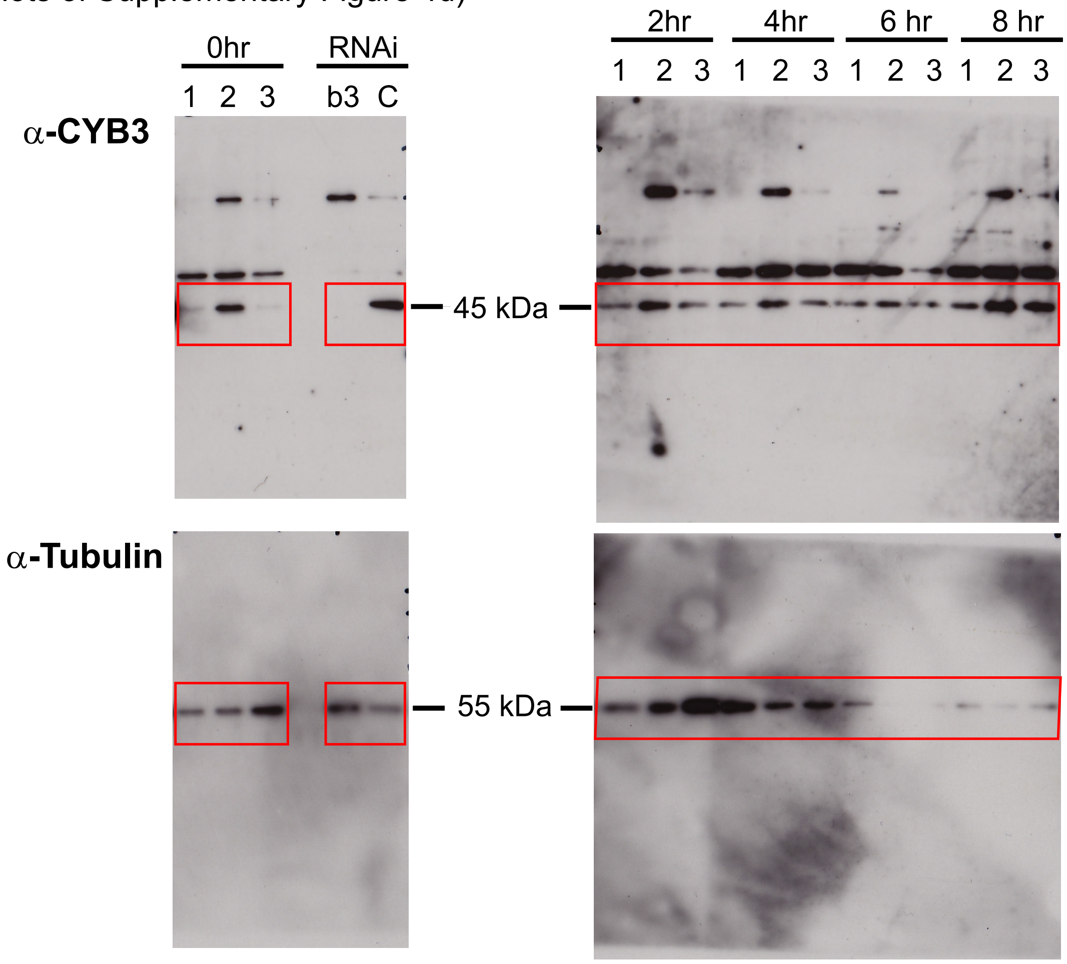

(blots of Supplementary Figure 4e)

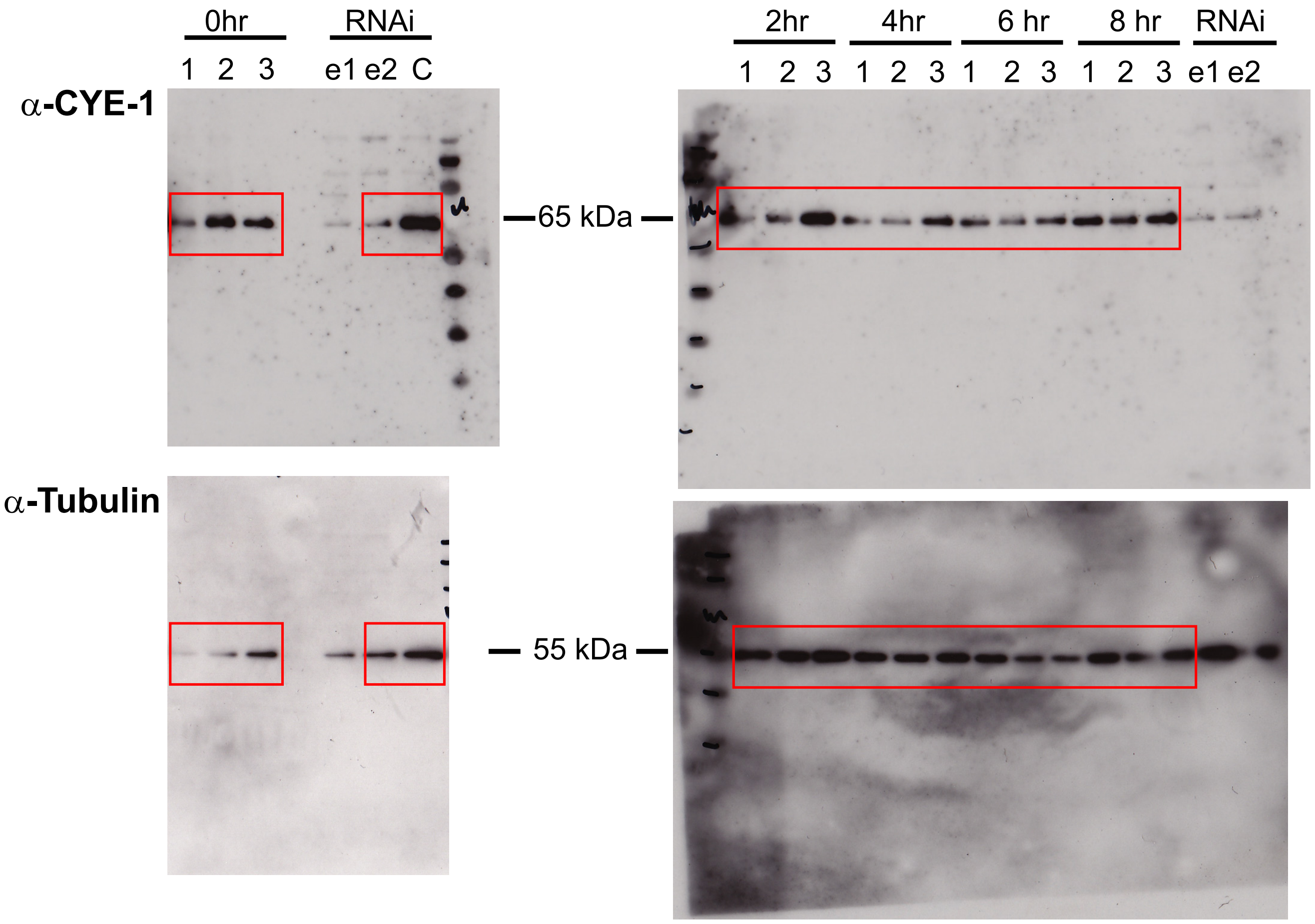

Supplementary Figure 6. Uncropped scans of gels and immunoblots. Boxes highlight the area selection used in the indicated figures.

Supplementary Table 1

Fosmid rescue of *fzr-1(he121)* as detected by reversion of *lin-35; he121 cyd-1* fertility and normal development

	**Rol animals**	**% fertile**	**% non-fertile**	**% wt vulva**	**% no vulva**	**% abnormal vulva**	**% n.d.**
Line 1 (n=110)	GFP+ (n=63)	1.6	98.4	0	70	17.4	12.7
	GFP- (n=47)	100	0	100	0	0	0
Line 2 (n=141)	GFP+ (n=60)	0	100	0	73.3	23.3	3.3
	GFP- (n=81)	96.3	3.7	60.5	0	1.2	38.3

Examination of Rol progeny from two balanced lines containing an extrachromosomal array with *myo-2::GFP* and a fosmid containing the *fzr-1* genomic region. Loss of fertility and normal development of *lin-35(RNAi); rol-1 he121 cyd-1* offspring correlates strongly with the presence of the extrachromosomal array. Animals with a wild-type vulva, no vulva or abnormal vulva (often protruding) are shown as percentages of the number of animals with or without pharyngeal GFP expression (indicating inheritance of the array). n.d.: % of animals not analysed for the vulval phenotype.

Supplementary Table 2
Soaking RNAi of *fzr-1* rescues the fertility of *lin-35; cyd-1* animals

**concentration *fzr-1(RNAi)* (ng per µl)**	**# fertile F1**	**# F2 progeny**
no RNAi	0	n.a.
50	3	20<br>6<br>6
500	2	23<br>6
1,000	1	17
2,000	1	24

For each concentration 6 animals were tested. The number of fertile F1 is indicated and for each fertile animal the number of viable F2 progeny.

Supplementary Table 3
Quantitative MS analysis of LIN-35, FZR-1 and FZR1 phosphorylation levels in kinase assays

C.elegans LIN-35 SEQUENCE	SITE	CDK-4 WT/ CYD-1	CDK-4 KD/ CYD-1	Ratio CDK-4 WT/KD
LNSYSPIKFTPIK	**S714**	9.72×10^6	Not Detected	Infinite
LNSYSPIKFTPIK	**T719**	6.89×10^6	Not Detected	Infinite
LNSYSPIKFTPIK	**S714, T719**	1.80×10^6	Not Detected	Infinite

C.elegans FZR-1 SEQUENCE	SITE	CDK-4 WT/ CYD-1	CDK-4 KD/ CYD-1	Ratio CDK-4 WT/KD
TLGPHNSPVKSMSTNSSAHTSPR	**S31**	9.41×10^9	1.72×10^8	54.7
TLGPHNSPVKSMSTNSSAHTSPR	**S31/S45**	6.51×10^7	Not Detected	Infinite
SMSTNSSAHTSPRVTPTK	**T49**	4.27×10^8	6.41×10^6	66.5
SMSTNSSAHTSPRVTPTK	**S45/T49**	2.17×10^9	9.64×10^5	2253.5
SMSTNSSAHTSPRVTPTK	**S41/S45/T49**	1.17×10^8	Not Detected	Infinite
SMSTNSSAHTSPR	**S45**	2.38×10^7	5.58×10^5	42.6
YHSINSDDDSGFK	**S77**	3.83×10^5	Not Detected	Infinite
ISGAESPTMAQMMEPR	**S275**	2.35×10^9	3.21×10^6	733.8
APPPALPLSPIVQK	**S320**	2.67×10^7	Not Detected	Infinite
APPPALPLSPIVQKQSPAR	**S320/S327**	2.16×10^7	Not Detected	Infinite
SLFTYSAKTTPVK	**S336/T340**	2.25×10^8	Not Detected	Infinite
SLFTYSAKTTPVK	**T340**	2.62×10^7	Not Detected	Infinite
SLFTYSAK	**Y335**	3.65×10^7	2.79×10^6	13.1
YGGQATTTATSPFGGPFGVDSQR	**S354**	3.84×10^6	Not Detected	Infinite

H. sapiens FZR1 SEQUENCE	SITE	CDK4 WT/ Cyclin D2	CDK4 KD/ Cyclin D2	Ratio CDK4 WT/KD
GSPNSMDQDYER	**"-S4"**	6.76×10^5	1.46×10^5	4.62
GSPNSMDQDYER	**Y5**	1.90×10^6	3.62×10^6	0.52
TLTPASSPVSSPSK	**T32**	2.35×10^7	6.24×10^6	3.77
TLTPASSPVSSPSK	**S36**	3.67×10^7	8.00×10^6	4.59
TLTPASSPVSSPSK	**S40**	1.06×10^7	3.18×10^6	3.35
SSPDDGNDVSPYSLSPVSNK	**S149**	8.61×10^5	1.08×10^6	0.79
SSPDDGNDVSPYSLSPVSNK	**S151**	4.54×10^6	Not detected	Infinite
SSPDDGNDVSPYSLSPVSNKSQK	**S157**	1.59×10^6	1.67×10^6	0.95

Phosphopeptides from LIN-35 and FZR-1 were identified by LC-MS/MS. Extracted ion chromatograms (AREAS) were obtained for each phosphopeptide in both the wild type and kinase dead (WT and KD) experiments. The intensities of all the phosphopeptides containing the same phosphosite(s) were summed to obtain a total signal in each experiment. The intensities for the different charge states of the same phosphopeptide (e.g. 2+, 3+, 4+...) were also summed.

Supplementary Table 4
Strain list

EMS mutagenesis
SV357: *lin-35(n745)/dpy-5(e61) unc-29(e91)* I *; rol-1(e91) cyd-1(he112)/mnC1[dpy-10(e128) unc-52(e444)]* II

whole genome sequencing
SV331: *lin-35(n745)* I*; rol-1(e91) cyd-1(he112)/mnC1[dpy-10(e128) unc-52(e444)]* II*; lin-15(n767)* X
SV383: *lin-35(n745)* I*; rol-1(e91) he121 cyd-1(he112)* II
SV789: *he121 rol-1(e91)* II*; cdk-4(gv3)* IV

***cdk-4* rescue experiments**
N2: wild type Bristol
SV913: *cdk-4(gv3)* X/+*; he Is22 [Pcdk-4::cdk-4KD:: flag::cdk-4 3'UTR]*
SV916: *cdk-4/*+ X
SV929: *unc-119(ed3)* III; *cdk-4(gv-3)X; vmIs9[unc-119; Pcdk-4::cdk-4::flag::cdk-4 3'UTR]*

brood size
N2: wild type Bristol
SV314: *rol-1(e91) cyd-1(he112)/mnC1[dpy-10(e128) unc-52(e444)]* II
SV216: *lin-35(n745)* I
SV1444: *lin-35(n2239)* I
SV439: *fzr-1(he121) rol-1(e91) cyd-1(he112)/mnC1[dpy-10(e128) unc-52(e444)]* II
SV981: *lin-35(n2239) I; fzr-1(he121) rol-1(e91) cyd-1(he112)* II
SV1303: *dpy-10 (e128) fzr-1(he121) rol-1(e91) cyd-1(he112)* II
SV1304: *dpy-10 (e 128) fzr-1(he121) rol-1(e91) cyd-1(he112)* II
SV789: *he121 rol-1(e91)* II*; cdk-4(gv3)* X *on lin-35(RNAi)*

***fzr-1(RNAi)* soaking**
SV1231: *lin-35; rol-1 cyd-1/mnC1; heSi45[Pmcm-4::mcm-4::mCherry::mcm-4 3'UTR; unc-119+]* IV

***fzr-1* fosmid expression**
SV1258: *fzr-1(he121) rol-1(e91) cyd-1(he112)/mnC1; heEx427[fosmid WRM0635dH03; Pmyo-2::gfp]*
SV1259: *fzr-1(he121) rol-1(e91) cyd-1(he112)/mnC1; heEx428[fosmid WRM0635dH03; Pmyo-2::gfp]*

egfp::fzr-1
SV1430: *fzr-1(ku298) unc-4(e120)* II*; heEx505[Plin-48::TdTomato; Pfzr-1::mEGFP::fzr-1::fzr-1 3'UTR]*
SV1454: *rol-1(e91) cyd-1(he112)/mIn1; heEx505[Plin-48::TdTomato; Pfzr-1::mEGFP::fzr-1::fzr-1 3'UTR]*

lin-35::gfp
SV1269: *unc-119(ed3)* III*; heSi45[Pmcm-4::mcm-4::mCherry]* IV*; heEx510[Plin-35::lin-35::GFP on fosmid wrm063cf08]*

cki::gfp
SV1275: *cki-1(gk132); heEx512[Pcki-1::cki-1::GFP on fosmid WRM0611ch10 ; Plin-48::TdTomato]*

S-phase reporter lines
SV1067: *unc-119(ed3)* III*; heSi45[Pmcm-4::mcm-4::mCherry::mcm-4 3'UTR; unc-119+]* IV
SV1263: *lin-35 (n745)/dpy-5(e61) unc-29 (e403); heSi73[Pmyo-3:: NLSegl-13::nzyfp]* II*; he-Si74[Pmcm-4:: NLSegl-13::czyfp]* IV*; lin-15A(n767)* X
SV1175: *fzr-1(ku298) unc-4(e120)* II*; unc-119(ed3)* III*; heSi45[Pmcm-4::mcm-4::mCherry::mcm-4 3'UTR; unc-119+]* IV
SV859: *heIs11[myo-3::H2B::GFP]*

Cyclin antibodies stainings and immunoblots
N2: wild type Bristol
MH1829*: fzr-1(ku298) unc-4(e120)* II
SV981: *lin-35(n2239) I; fzr-1(he121) rol-1(e91) cyd-1(he112)* II

Supplementary Table 5
Primers, oligos and siRNAs

Name	Sequence
FLAG tag linker F	AATTCCGATTACAAGGATGATGATGATAAGTGAGGATCC
FLAG tag linker R	AATTGGATCCTCACTTATCATCATCATCCTTGTAATCGG
*Not*I linker	GTACGCGGCCGC
Asp187Asn F	CGTGAAATTGGCAAATTTTGGATTGTCAAAAG
Asp187Asn R	CTTTTGACAATCCAAAATTTGCCAATTTCACG
NLSegl-13	GTCGACAAAAAATGAGCCGTAGACGAAAAGCGAATCCGACAAAACTGAG-TGAAAACGCGAAGAAACTTGCCAAGGAAGTTGAAAATA AGGTACC
*mcm-4*prom F	TCTAGACATCCACGTCATCATCC
*mcm-4*prom R	CTAGCTGCAAAAATTTACAGATTTCGC
lin-35 fosmid F	CGAACCTTGAAAAATCTGGAATTACGATCGCTATGGAACGGTCTGGA-GATGGAGGAGGATCTGGAGGAGGAGGATCTGGAGGAGGA
lin-35 fosmid R	GATTAATCAAAAATGCATTAAAAAGTTCAAAGTATTCACAACAATCATTTT-TATTCATGCCATTCAATCTTCTGAGCTTCG
cki-1 fosmid F	CAAAATCTCGTCGTCCAAC-GATCAGAACTCGATCTTCATGCTCTCCATACGGAGGAGGATCTGGAG-GAGGAGGATCTGGAGGAGGA
cki-1 fosmid R	GATTAGATTCAACCCGTGGAATTGGGCAAAAAAAAGTCAAAATGAAAC-CTCTATTCATGCCATTCAATCTTCTGAGCTTCG
*cdk-4 Xho*I_5' F	CCGCTCGAGCCACCATGTGCGAGAATCTTTATG
cdk-4 _1026_*Hd*III R	CCCAAGCTTCTTGTTGAAGTTGATTTGCT
cdk-4KD	TCAACAGAGATCAAACCGTGAAATTGGCAAATTTTGGATTGTCAAAAGAG-TACTC
3xFLAG_*Hd*III F	CCCAAGCTTATGGACTACAAAGACCATGAC
3xFLAG_*Pst*I R	CATTGGTTCTGCAGCTACTTGTCATCGTCATCCTT
H.s._cdk-4_FLAG G-block	CTCGAGATGTATCCCTGCCCCGTG-GAGCCTTTCCCCCCAGAGGGCCCCGCCCAGTGCAGTCGGTGGTAC-CTGAGATGGAGGAGTCGGGAGCACAGCTGCTGCTG-GAAATGCTGACTTTTAACCCACACAA-GCGAATCTCTGCCTTTCGAGCTCTGCAGCAC-TCTTATCTACATAAGGATGAAGGTAATCCGGAGGACTACAAAGAC-CATGACGGTGATTATAAAGATCATGACATCGATTACAAGGATGAC-GATGACAAGTGAGCAATGGATCCGTCGAGGAATTCACTCCTCAGGTG-CAGGCTGCCTATCAGAAGGTGGTGGCTGGTGTGGCCAATGCCCTGG
*cyd-1*_1 *Eco*RI_F	GGAATTCGGCACCATGGACTTTGAGTCG
*cyd-1*_1218 *Sal*I_R	ACGCGTCGACTCATAAAGTCTTGAAGATCTTCGG
*fzr-1*_1 *Sal*I-F	ACGCGTCGACATGGATGAGCAGCAACCGCC
*fzr-1*_1197 *Not*I-R	ATAAGAATGCGGCCGCATTCTGTGATGACCAATCGACC)
*fzr-1*_988 *Eco*RI-F	GCGGAATTCCCGATCACTTTTTACGTACAGTGC
*fzr-1*_2107 *Not*I-R	ATAAGAATGCGGCCGCCCGGATGGTAGAATGCAAATTC
*lin-35*_1486 *Sma*I-F	TGCCCCGGGGACGATTTGGAATCGAACAAT
*lin-35*_2883_*Not*I-R	ATA AGA ATG CGG CCG CAT CTC CAG ACC GTT CCA TAG
*fzr1_Hs_Eco*RI_1 F	GCGGAATTCCATGGACCAGGACTATGAGC
*fzr1_Hs_Not*I_598 R	ATAAGAATGCGGCCGCATTGAGGGACGACCAGTCCA
siControl	CGUACGCGGAAUACUUCGA
FZR1 siRNA#1	CCACAGGAUUAACGAGAAU
FZR1 siRNA#2	GGAACACGCUGACAGGACA
FZR1 siRNA#3	GCAACGAUGUGUCUCCCUA
FZR1 siRNA#4	GAAGAAGGGUCUGUUCACG
Rb siRNA#1	AAACUACGCUUUGAUAUUG
Rb siRNA#2	GAAUCUGCUUGUCCUCUUA
Rb siRNA#3	CGAAAUUGGAUCACAGCGA
Rb siRNA#4	GGUUCAACUACGCGUGUAA
